# Substitute Yeast Extract While Maintaining Performance: Showcase Amorpha‐4,11‐Diene Production

**DOI:** 10.1111/1751-7915.70056

**Published:** 2024-11-21

**Authors:** Carlos Castillo‐Saldarriaga, Christine N. S. Santos, Stephen Sarria, Parayil K. Ajikumar, Ralf Takors

**Affiliations:** ^1^ Institute of Biochemical Engineering University of Stuttgart Stuttgart Germany; ^2^ ManusBio Cambridge Massachusetts USA

**Keywords:** amino acids, complex media, *Escherichia coli*, fermentation, pyruvate, terpenoids

## Abstract

Yeast extract (YE) is a complex nutritional source associated with high performance on microbial production processes. However, its inherent compositional variability challenges its scalability. While prior efforts have focused on growth‐associated products, the dynamics of growth‐uncoupled production, which leads to higher production rates and conversion yields, still need to be explored. This production scenario is common in large‐scale applications. This study presents a systematic approach to replace YE for the production of the terpene amorpha‐4,11‐diene in *Escherichia coli* . Sequential processing was successfully applied to identify glutamic acid, alanine, leucine, valine, isoleucine and glycine as the key amino acids (AAs) under slow‐growth conditions. Thoroughly applying biomass retention as part of sequential processing increased production capacity by 45% using these AAs instead of YE. Further studies, including flux balance analyses, targeted pyruvate as the common AA precursor. The optimized fed‐batch process feeding pyruvate with 0.09 g_Pyr_ h^−1^ enhanced amorpha‐4,11‐diene production by 37%, although adding only 1% carbon via pyruvate. Flux balance analysis revealed the criteria for optimum pyruvate feeding, for example, to prevent succinate secretion and maintain the NADH/NAD^+^ balance. These findings illustrate the interplay between media composition and metabolic activity and provide a successful guideline for identifying lean, best‐performing media for industrial applications.

## Graphical abstract

We investigated AA requirements for an AMD4,11‐producing *Escherichia coli* strain to replace yeast extract (YE) under decoupled‐growth production conditions. By substituting YE with specific AAs and feeding a metabolic precursor, production capacity increased by up to 45%.

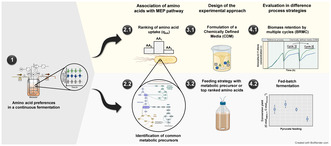

## 1 Introduction

Yeast extract (YE) is widely used in media formulations to provide multiple essential nutrients for microbial production. Although its use is advantageous for enhancing strain performance, the inherent variability in YE composition threatens the reproducibility of processes on a large scale and hinders the comprehensive understanding of strain robustness (Jacob et al. 2019b; Tao et al. 2023). For instance, discrepancies in composition between lots or brands lead to a 50% of variation in the final cell dry weight (CDW) and specific growth rates, even without considering the strain production capacity (Potvina et al. 1997).

Previous studies have demonstrated that the proteinogenic composition of YE is associated with strain phenotype, media, production conditions and disruption methods (Jacob, Hutzler, and Methner 2019; Jacob et al. 2019c). Therefore, numerous efforts have been made to elucidate the interactions between YE components and metabolic responses, particularly in *Escherichia coli* , a standard workhorse in the academic and industrial sectors. Nancib, Branlant, and Boudrant (1991) found that YE enables acetate assimilation and growth of *E. coli* cells during recombinant protein production. Similarly, Diederichs et al. (2014), reported significant differences in metabolic activity and product yield associated with different YE lots and brands in recombinant *E. coli* variants. Data analysis and modelling tools have also been implemented to enrich the understanding of these interactions. Zampieri et al. (2019) developed a constraint‐based model that integrated nontargeted exometabolomics to provide insights into the decision‐making process of *E. coli* during the transition from complex to minimal media. Furthermore, Tachibana, Watanabe, and Konishi (2019) conducted a multivariate data analysis to correlate the YE composition and growth performance of *E. coli* to provide a toolbox for optimizing cultivation and quality control.

Remarkably, efforts have been made to understand the role of free amino acids (AAs) in YE as metabolome‐reprogramming metabolites that improve cellular energy and carbon efficiency (Bodini, Nunziangeli, and Santori 2007; Selvarasu et al. 2009; Cheng et al. 2019). Chow et al. (2006) found that the consumption of Asn, aspartic acid (Asp), Gln and glutamic acid (Glu) enhances the production of elastin‐like proteins and biopolymers enriched in glycine (Gly), valine (Val), proline (Pro) and alanine (Ala). This illustrates that, in some cases, there is no direct association between the most consumed AAs and the final product composition. Tachibana, Chiou, and Konishi (2021) applied a deep neuronal network‐mediated metabolomic approach to identify Glu, Ala, Phe, isoleucine (Ile), Lys and Asp as essential components for cell growth, emphasizing the significance of Glu and Asp in increasing the GFP yield in *E. coli* . Most studies have focused on the association between AA consumption and growth‐related products. This excludes uncoupled growth production processes, which may anticipate the highest conversion yields owing to the prevention of carbon utilization for growth. Currently, multiple metabolites such as terpenes and flavonoids are produced using this principle.

Amorpha‐4,11‐diene (AMD4,11), a sesquiterpene, is frequently employed as a proof‐of‐concept molecule due to its academic and industrial relevance as a key intermediate in the biosynthesis of the antimalarial artemisinin. Importantly, the main goal in microbial production of AMD4,11 and other terpenes is to decouple growth from production, reducing carbon loss to biomass formation (Patil et al. 2021; Castillo‐Saldarriaga et al. 2024). This is particularly important if the methylerythritol pathway (MEP), which consumes pyruvate, is used to synthesize isoprenoid precursors for terpene production, dimethylallyl pyrophosphate (DMAPP) and isopentyl pyrophosphate (IPP). Consequently, nongrowth‐coupled production increases the required supply of ATP and NADPH, which are not regenerated in the MEP pathway (Yang et al. 2016; Clomburg et al. 2019).

Therefore, this study investigated AA requirements of AMD4,11‐producing *E. coli* strain under minimum growth rate conditions to replace YE, a fermentation enhancer, while maintaining performance. The sesquiterpene is accessed through the MEP pathway in a continuous process that is supplemented with YE. AA consumption rates were ranked to identify common metabolic precursors. Consequently, two concepts were evaluated: (1) replacing YE with a selected group of AAs to execute multiple cycles in the biomass retention process and (2) feeding the metabolic precursor to stimulate AMD4,11 production. Both concepts were successfully implemented, resulting in a mass‐based increased in AMD4,11 production of up to 45%.

## 2 Experimental Procedures

### 2.1 Strain

*E. coli* AMD4,11 strain was provided by Manus Bio. This strain, derived from *E. coli* MG1655, was engineered to increase the carbon flux through the MEP pathway for producing isoprenoid precursors IPP and DMAPP (Kumaran et al. 2022). The phosphoglucose isomerate gene (*pgi*) was also knocked out and the AMD4,11 synthase gene derived from *Artemisia annua* was integrated to produce AMD4,11.

### 2.2 Media and Growth Conditions for Seed Cultivation

Seed cultures for bioreactors were prepared by inoculating 500 mL of batch medium with 1 mL of frozen cell stock (30% w v^−1^ glycerol) of *E. coli* AMD4,11 strain. The medium composition and seed cultivation conditions have been described previously (Patil et al. 2021). Seed cultures were harvested during exponential growth at an optical density of approximately 3–4 before inoculating the bioreactors. The inoculum volume was 6% (v v^−1^) of the bioreactor working volume.

### 2.3 Fermentation Media, Feeding Solutions and Bioreactor Set‐Up

For the ‘sequential’ process, biomass retention by multiple cycles (BRMC), and fed‐batch fermentation experiments, the batch fermentation media composition consisted of glucose (9.3 g L^−1^), ammonium sulfate ((NH_4_)_2_SO_4_; 4 g L^−1^), potassium phosphate monobasic (KH_2_PO_4_; 13.33 g L^−1^), potassium phosphate dibasic (K_2_HPO_4_; 5.09 g L^−1^), citric acid (C_6_H_8_O_7_; 1.11 g L^−1^), YE (22.21 g L^−1^), iron (II) sulfate heptahydrate (FeSO_4_·7H_2_O; 44.47 mg L^−1^), magnesium sulfate (MgSO_4_; 333.6 mg L^−1^), thiamine hydrochloride (5 mg L^−1^), trace element solution (1.22 mL L^−1^) and antifoam agent (Struktol J647; 0.61 mL L^−1^). Trace element solution contained manganese (II) chloride tetrahydrate (MnCl_2_·4H_2_O; 10 g L^−1^), zinc sulfate heptahydrate (ZnSO_4_·7H_2_O; 3.2 g L^−1^), copper (II) chloride dihydrate (CuCl_2_·4H_2_O; 1 g L^−1^), calcium chloride dihydrate (CaCl_2_·2H_2_O; 40 g L^−1^), boric acid (H_3_BO_3_, 0.5 g L^−1^) and sodium molybdate dihydrate (NaMoO_4_·2H_2_O; 2 g L^−1^). Before inoculation, the pH of the medium was adjusted to 7 using 6 N NaOH.

For the fed‐batch and BRMC experiments, the carbon‐feeding solution composition was 545 g L^−1^ glucose, 30.6 g L^−1^ KH_2_PO_4_, 36.6 g L^−1^ K_2_HPO_4_, 7.42 mg L^−1^ thiamine hydrochloride and 1.6 mL L^−1^ trace element solution. The nitrogen‐feeding solution contained 99 g L^−1^ (NH_4_)_2_SO_4_ and 18.75 mL L^−1^ of 25% NH_4_OH. Solutions of 6 N NH_4_OH and 6 N NaOH were used to control the pH. In the case of continuous stage of the sequential process, the carbon‐feeding composition was adjusted to 372 g L^−1^ glucose, 13.79 g L^−1^ KH_2_PO_4_, 16.5 g L^−1^ K_2_HPO_4_, 20 g L^−1^ YE (as an organic source of free AAs), 5.02 mg L^−1^ thiamine hydrochloride and 1.082 mL L^−1^ of trace element solution. The nitrogen feeding and pH solutions had compositions similar to those used for the fed‐batch and BRMC experiments.

All the experiments were performed in a DASGIP parallel bioreactor system equipped with a GA4 module for off‐gas analysis (Blue Sens) and an MP8 module with previously calibrated peristaltic pumps. Each bioreactor vessel was fitted with three six‐blade Rushton‐type impellers and multiple‐dip tubes for sampling and harvesting. pH and dissolved oxygen were monitored using probes from Mettler Toledo (405‐DPAS‐SCK8S) and Hamilton (VisiFerm DO ECS 325), respectively.

Regardless of the fermentation experiment, the temperature was maintained at 37°C. A pH set point of 7 was controlled automatically using 6 N NH_4_OH, and the base was switched to 6 N NaOH after adding 180 mmol of NH_4_OH. The nitrogen feed was into the process to maintain ammonium levels between 30 and 60 Mm, with the volumetric flow rate adjusted as needed across all experiments (Patil et al. 2021). Dissolved oxygen was maintained at 40% during the first 24 h, with an agitation cascade starting at 300 rpm. The aeration rate was constantly maintained at 12 L h^−1^. For exclusive use in the fed‐batch process, a 10% (w v^−1^) safflower oil extraction phase was used.

### 2.4 Identification of AA Preferences Under Low‐Growth Conditions by Applying a Sequential Process

Two distinct stages were employed in the sequential process, followed by a batch phase. The initial stage utilised a stainless‐steel probe with 0.22 μm polypropylene membrane for in situ filtration. This probe functioned as a biomass retention unit, facilitating the constant harvesting of cell‐free fermented broth, while maintaining carbon limitations. Subsequently, the biomass retention unit was deactivated in the second stage and a continuous dilution rate of 0.01 h^−1^ was implemented. This phase involved a dip tube adjusted to the height of the stirred liquid surface in the reactor to ensure continuous harvesting of the fermented broth. The volumetric flow rate was monitored and gravimetrically adjusted every 2–3 h. The entire process lasted for approximately 140 h.

During the second stage, when a continuous process was established, the carbon‐feeding composition was adjusted as described in Section 2.3, incorporating YE as an exogenous source of AAs. YE composition is shown in Table S1. Samples were collected for the analysis of CDW, glucose, AAs and AMD4,11 concentrations. AA uptake rates were calculated and organized using analysis of variance (ANOVA) with Tukey's post hoc test.

### 2.5 Evaluation of Supplementation Strategies

**FIGURE 1:**
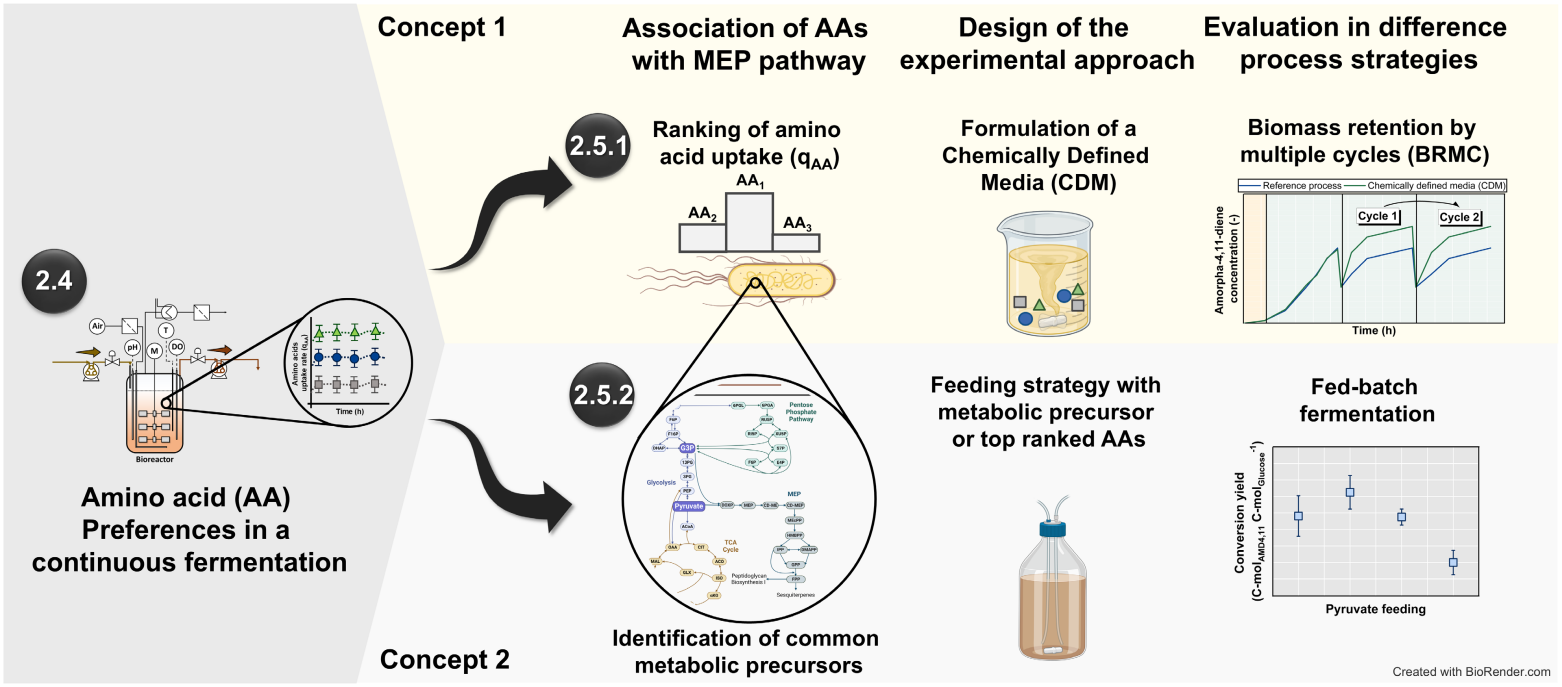
Comprehensive methodology for transitioning from a complex production media through batch media reformulation and supplementation of metabolic precursors. The numerical labels correspond to the Experimental Procedure Section.

Two concepts were applied to study the need for and supplementation of AAs (Figure 1). Concept 1 identified and tested AAs with the highest uptake rates in the BRMC mode. Concept 2 focused on feeding pyruvate, which was revealed as a common precursor for the top‐ranked AAs in Concept 1. Fed‐batch experiments were also conducted.

#### 2.5.1 Concept 1: Evaluation of Top‐Ranked AA Uptake Rates in BRMC Fermentations

BRMC allows high cell densities, supports production at low specific growth rates and facilitates the concentration of product inside the bioreactor during production. These features provide unique advantages over traditional systems, such as batch or fed‐batch processes (Castillo‐Saldarriaga et al. 2024). Therefore, top‐ranked AA uptake rates were evaluated using this strategy. After 54 h of BRMC fermentation, carbon feed was stopped and 50% of the retained fermenter content was replaced with fresh medium. Samples were collected before and after the exchange. Subsequently, the BRMC mode was reactivated. After 98 h, the medium exchange was repeated, and BRMC was restarted. An antifoam was added during the second cycle to control foaming. All exchanged masses were measured gravimetrically.

BRMC tests considered three different replacement media: (a) BRMC‐Control mirrored the batch medium with doubled concentrations of vitamins, trace elements and YE to reinstall original batch concentration after the dilution replacement step (See Section 2.3), (b) BRMC‐MixAA substituted 40 g/L YE with the equivalent amount of the top‐ranked AAs (L‐Glutamic acid, L‐Alanine, L‐Leucine, L‐Valine, L‐Isoleucine and L‐Glycine), (c) BRMC‐w/oAA neither considered YE nor additional AAs. Samples were collected as described in the previous sections. The media composition can be found in Table S2.

#### 2.5.2 Concept 2: Substituting AAs via Pyruvate Feeding in a Fed‐Batch Process

Pyruvate was fed during the stationary phase of a fed‐batch process using three mass flow rates: 0.09 (F1), 0.18 (F2) and 0.27 (F3) g_Pyr_ h^−1^. The pyruvate concentration in stock solutions was adjusted to achieve accurate mass feeding rates, minimising variations in volumetric feeding rates across treatments. The control tests did not include pyruvate supplementation. Sampling was intensified to measure AMP, ADP, ATP, NAD^+^ and NADP^+^ levels. Detailed information about medium composition and fermentation parameters can be found in Section 2.3.

Constraint‐based reconstruction and analysis was performed using the COBRA toolbox to elucidate the effects of pyruvate supplementation. Flux balance analysis (FBA) considered the objective function ‘ATP4sr’ to maximize ATP synthesis in the BiGG *E. coli* iJR904 model (Feist et al. 2007) using the GLPK solver. To cope with the strain genotype (Patil et al. 2021), the original model was adapted, as indicated in Supporting Information. The solution space was constrained by the measured uptake rates for glucose (*q*_Glucose_), oxygen ($q_{O_{2}}$) and pyruvate (*q*_Pyr_), the specific growth rate (*μ*) and AMD4,11 production (*q*_AMD4,11_).

The conversion yields and batch productivity were calculated using Equation (1) and (2), respectively.

(1) YPS=mPmGlucose

(2) $$Q_{P}=\frac{m_{P}}{t_{P}}$$

$\text{where}\;m_{P}$ represents the total amount of AMD4,11 (mg), $m_{\text{Glucose}}$ is the total amount of glucose consumed, and $t_{P}$ is the effective processing time. For BRMC, $m_{P}$ considered all products containing volume fractions. For pyruvate analysis, conversion yields were calculated on a C‐mol basis to evaluate the carbon economy, while glucose was co‐consumed.

### 2.6 Analytical Procedures

#### 2.6.1 CDW Concentration

For CDW (g_CDW_ L^−1^) determination, the biomass pellet was obtained by centrifugation of 2–3 mL of the sample at 4615 rpm (2500 rcf) and 4°C for 7.5 min (5430R, Eppendorf). The supernatant was recovered for offline measurements, and the biomass pellet was washed twice with 0.9% (w v^−1^) NaCl solution. Subsequently, the pellet was dried for at least 24 h at 105°C, and the CDW was calculated gravimetrically.

#### 2.6.2 Glucose, Pyruvate, Organic Acids, Ammonium and Osmolality Determination

The supernatant obtained from the CDW concentration step was filtered through 0.22 μm cellulose acetate filters and stored at −70°C for further analysis. The samples were diluted in deionized water to determine glucose to achieve a theoretical concentration between 0.4 and0.5 g_Glucose_ L^−1^. Twenty microliters of the diluted supernatant were added to the reaction cups of a LaboTrace analyzer (Trace Analytics).

Pyruvate (> 99%, Sigma‐Aldrich) and organic acids, such as acetate (99.8%, Sigma‐Aldrich), succinate (99.5%, Sigma‐Aldrich) and lactate (99%, Sigma‐Aldrich), were quantified using HPLC coupled with an RI detector (1100 series, Agilent) and a Hi‐Plex H column (Agilent Technologies). Owing to the presence of phosphate salts in the samples, a phosphate precipitation protocol was employed before injection. Details of the HPLC method and sample preparation can be found in a previous study (Michalowski, Siemann‐Herzberg, and Takors 2017).

Ammonium ion concentration was monitored during the fermentation experiments using a colorimetric kit (LCK 303, Hach Lange GmbH). Preliminary tests were conducted to rule out any interference caused by YE in the medium. Moreover, since sodium pyruvate was used as a pyruvate source, an osmometer (osmomat 030, Gonotec) was used to check the osmolality levels in all the replicates.

#### 2.6.3 AA and Nucleotide Analysis

The concentration of AA was determined using HPLC coupled with a diode array (DAD) and fluorescence detector (1200 series, Agilent) and an Agilent Zorbax Eclipse Plus C18 column with a precolumn (250 × 4.6 mm, 5 μm). The differential dilution factors of the supernatant samples were fixed to ensure the proper quantification of each AA. A standard AA mix (AAS18, Sigma‐Aldrich), in addition to tryptophan, glutamine and asparagine, was used to construct the calibration curve. 4‐aminobutanoic acid (GABA) was used as internal standard. Specific details of the quantification method have been reported elsewhere. (Ulmer et al. 2022).

For nucleotide analysis, 2 mL of fermented broth with microbial biomass was sampled from the bioreactor directly into 0.5 mL of precooled quenching solution (80 μM EDTA dissolved in 35% (v v^−1^) perchloric acid) and incubated at 6°C on a rocking mixer for 15 min. Neutralization of the quenched samples was performed as previously described (Ziegler et al. 2021). An HPLC coupled with a DAD and a Thermo Scientific BDS Hypersil C18 column with a precolumn (150 × 4.6 mm, 3 μm) was used for the determination of AMP, ADP, ATP, NAD^+^ and NADP^+^. A single calibration curve with all standards was developed and guanosine 3, 5‐cyclic monophosphate was included as an internal standard. HPLC quantification and data analysis were performed as described previously (Löffler et al. 2016). The AxP values were used to calculate the adenylate energy charge (AEC, Equation (3)) described by (Atkinson and Walton 1967).

(3) $$\mathrm{AEC}=\frac{\left[\mathrm{ATP}\right]+0.5\left[\mathrm{ADP}\right]}{\left[\mathrm{AMP}\right]+\left[\mathrm{ADP}\right]+\left[\mathrm{ATP}\right]}$$

#### 2.6.4 AMD4,11 Extraction and Quantification

To extract AMD4,11, 400 μL of the sample from the bioreactor was transferred into glass tubes and mixed with 1600 μL of ethyl acetate (> 99.5%, Carl‐Roth) by vortexing at the maximum speed for 15 min. The mixture was centrifuged at 12,000 rpm and 4°C for 15 min, and the supernatant (ethyl acetate plus AMD4,11) was stored at −70°C for further analysis. The same extraction process was used on the permeate samples to validate the previously reported concentration effect due to membrane implementation (Castillo‐Saldarriaga et al. 2024).

Quantification of AMD4,11 was performed using a gas chromatography Agilent 5840 GC coupled with a flame ionization detector (FID) and Rxi‐5Sil MS column (Restek). The inlet, FID temperature, oven program and further details regarding the analytical method have been previously reported (Patil et al. 2021). Samples were obtained from biological replicates. Standard runs were included in between samples to ensure quantification consistency. Manus Bio provided the AMD4,11 standard for the analysis.

### 2.7 Statistical Information

All statistical data were obtained in triplicate, unless otherwise noted in the text or figure legends. The error bars represent one standard deviation of the mean. Statistical significance (*p* ≤ 0.05; *p* ≤ 0.01; and *p* ≤ 0.001) was determined by the Tukey post hoc test via one‐way ANOVA.

## 3 Results

### 3.1 Top‐Ranked AA Needs Identified in the Sequential Process

To identify the primary AAs utilized under slow‐growing conditions, we employed a sequential process including a batch, biomass retention and continuous phase (See Section 2.4). During the continuous phase, carbon and yeast extract were fed to the bioreactor.

**FIGURE 2:**
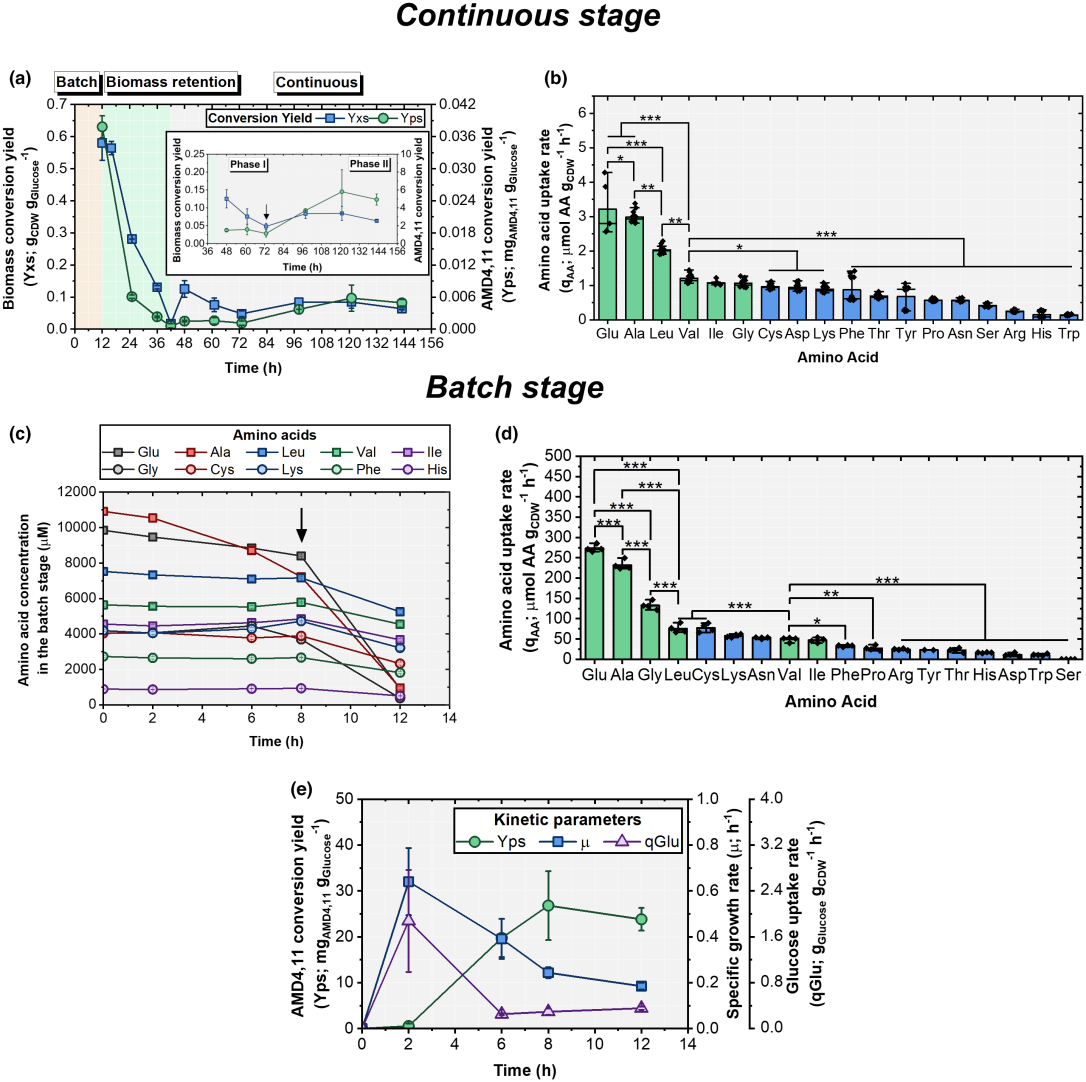
For the continuous stage of the sequential process: (a) Profiles of conversion yield of glucose to biomass (*Y*_XS_; g_CDW_ g_Glucose_^−1^) and amorpha‐4,11‐diene (*Y*_PS_; mg_AMD4,11_ g_Glucose_^−1^), (b) Mean AA uptake rate (*q*_AA_; μmol g_CDW_^−1^ h^−1^) with a dilution rate of 0.01 h^−1^; for the batch stage: (c) AA concentration (μM), (d) Mean AA uptake rate during the last 4 h of batch stage, and (e) AMD4,11 conversion yield, specific growth rate and glucose uptake rate. Data are represented as mean ± SD (*n* = 2 for a, *n* = 5 for glutamic acid; *n* = 8 for isoleucine, and *n* = 13 for the rest AAs in (b), and *n* = 4 for (c–e). For (a), the light yellow background indicates the batch phase, light green indicates the biomass retention phase, and light grey indicates the multiple‐cycle phase. For (b, d), asterisks indicate statistically significant differences (*p ≤ 0.05; **p ≤ 0.01; ***p ≤ 0.001) within the groups determined by ANOVA with Tukey's post hoc test. No asterisks indicate the absence of statistical significance. Green bars indicate the top‐ranked amino acids and blue the rest. In (c), the black arrow labels the start of AA consumption (see legend).

Two distinct phases were identified (Figure 2a): from 48 to 72 h, the substrate‐to‐biomass conversion yield *Y*_XS_ reduced by 61.6%, although *Y*_PS_ remained low at 1.40 ± 0.24 mg_AMD4,11_ g_Glucose_^−1^. After 72 h, *Y*_XS_ increased by 1.8‐fold to 0.08 ± 0.01 g_CDW_ g_Glucose_^−1^, which was still below the level of the precontinuous periods. However, *Y*_PS_ increased by 3.4‐fold compared with that in the initial continuous phase (see also Figure S1).

Analysis of the YE‐containing feed of the continuous phase led to statistically sound identification of individual uptake rates (Figure 2b). Glu, Ala and leucine (Leu) were the most abundant, followed by a group comprising Val, Ile and Gly. Consequently, all were grouped, covering uptake rates from 3.22 ± 0.80 to 1.06 ± 0.11 μmol g_CDW_^−1^ h^−1^.

A similar analysis was conducted for the preceding batch phase (Figure 2c,d) to determine the optimum growth conditions. Glucose uptake, AMD4,11 production rate and specific growth rates are shown in Figure 2e. Arginine (Arg), Asp, Pro, serine (Ser) and threonine (Thr) dominated AA uptake during the first 8 h, whereas Glu, Ala, Gly and Leu showed highest uptake rates afterwards. Maximum rates of 273.4 ± 8.7 and 232 ± 11.8 μmol g_CDW_^−1^ h^−1^ were reached for Glu and Ala, respectively. Further details are provided in Figures S1 and S2.

### 3.2 Simplifying Nutritional Complexity: A BRMC Concept With AAs as Substitutes

The top‐ranked AA identified from slow‐growing cells in the continuous phase of the sequential process were evaluated as potential YE substitutes. This evaluation was conducted in a BRMC process, known for its high productivity in AMD4,11 production (Castillo‐Saldarriaga et al. 2024).

**FIGURE 3:**
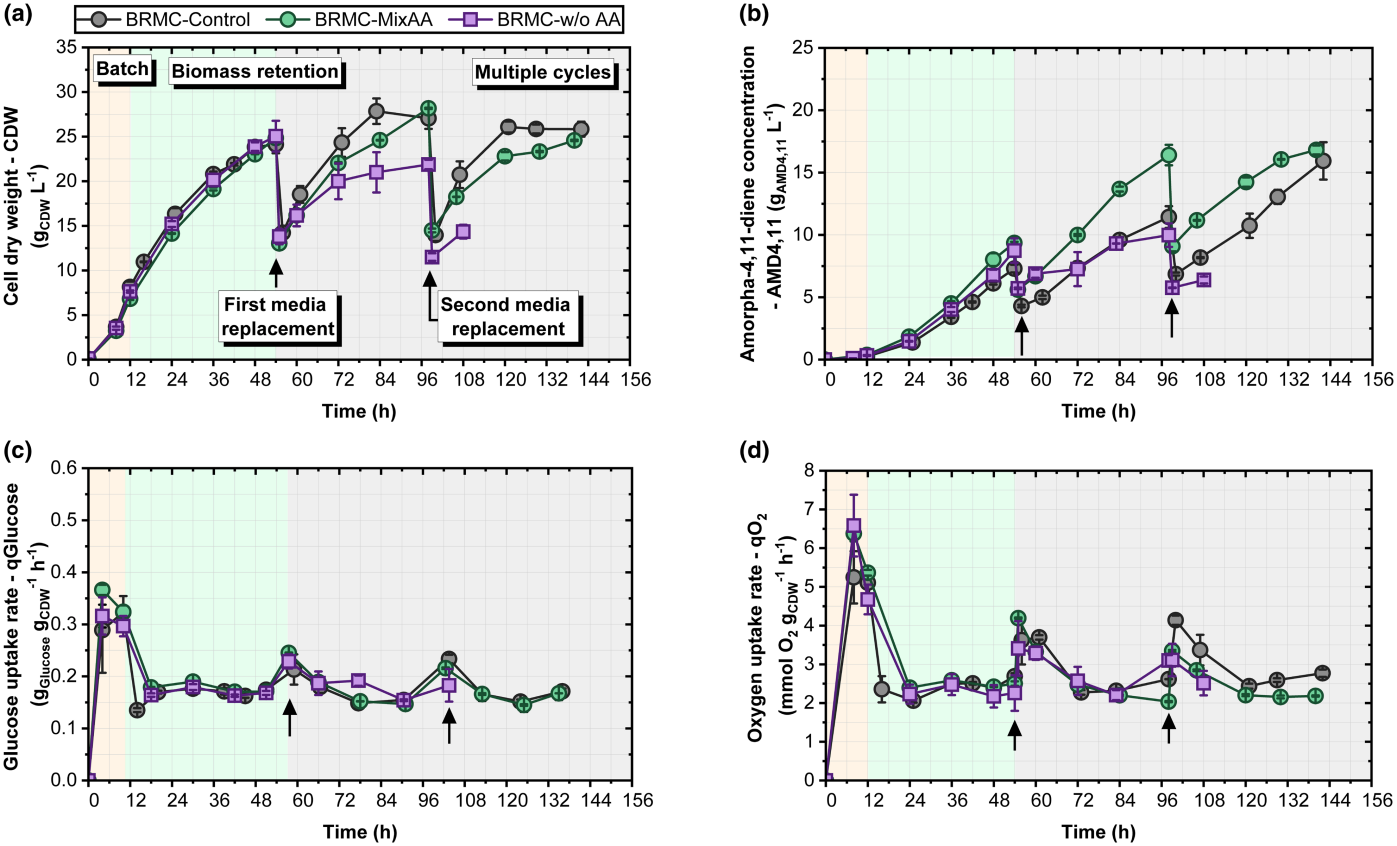
(a) Cell dry weight (CDW; g_CDW_ L^−1^), (b) amorpha‐4,11‐diene concentration (AMD4,11; g_AMD4,11_ L^−1^), (c) glucose uptake rate (*q*_Glu_; g_Glucose_ g_CDW_^−1^ h^−1^), (d) oxygen uptake rate ($q_{O_{2}}$; mmol O_2_ g_CDW_^−1^ h^−1^) and (d) amorpha‐4,11‐diene production rate (mg_AMD4,11_ g_CDW_^−1^ h^−1^) during the fermentation course using a biomass retention by multiple cycles strategy (BRMC) with different replacement media. The light yellow background indicates the batch stage, light green indicates the biomass retention stage, and light grey indicates the multiple‐cycle stage. Arrows indicate medium replacement. Data are presented as mean ± SD (*n* = 2).

As depicted in Figure 3, three different replacement media were evaluated in the BRMC mode: YE in BRMC‐Control, top‐ranked AAs in BRMC‐MixAA and without YE and any exogenous AAs in BRMC‐w/o AA. No growth differences were observed during batch or biomass retention (Figure 3a). The media exchange after 54 h let to similar biomass concentration of 27.6 ± 0.8 g_CDW_ L^−1^ for BRMC‐Control and BRMC‐MixAA, whereas BRMC‐w/o AA showed 20.8% less CDW. Partial replacement of YE by the top‐ranked AAs did not show significant differences respect to BRMC‐MixAA (Figure S3). Ammonium concentrations were similar in all three media (Figure S3). After the second media replacement, the same trend continued, leading to the discontinuation of BRMC‐w/o AA because of glucose accumulation.

Interestingly, the trends in AMD4,11 titers differed from those of cell growth, as BRMC‐MixAA outperformed the other media (Figure 3b). After 98 h, BRMC‐MixAA enabled 43.5% more AMD4,11 production than that of the control. Notably, the biomass concentrations were the same. During the second cycle, the differences between BRMC‐Control and BRMC‐MixAA diminished, whereas BRMC‐w/o AA maintained poor performance.

The rates of biomass‐specific glucose and oxygen uptake were comparable and stable in all settings (Figure 3c,d). Only BRMC‐w/o AA showed a slow glucose uptake in the late phase.

**FIGURE 4:**
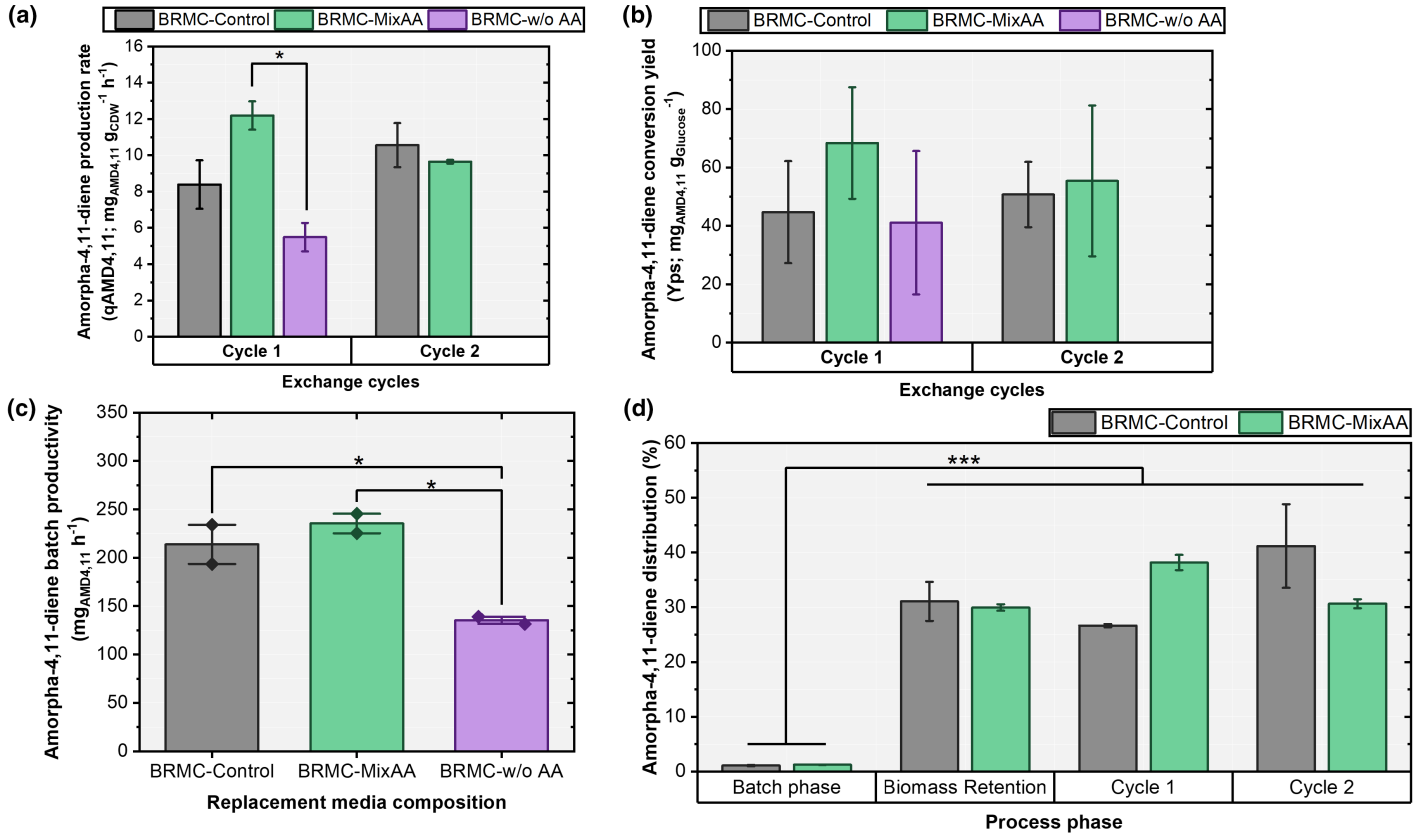
Amorpha‐4,11‐diene (a) production rate (*q*_AMD4,11_; mg_AMD4,11_ g_CDW_^−1^ h^−1^) in different cycles, (b) conversion yield (*Y*ps; mg_AMD4,11_ g_Glucose_^−1^), (c) bath productivity (mg_AMD4,11_ h^−1^) and (d) distribution among the process stages during the BRMC using different composition of the replacement media. Data are presented as mean ± SD (*n* = 2). Asterisks indicate statistically significant differences (*p ≤ 0.05; **p ≤ 0.01; ***p ≤ 0.001) within the groups determined by ANOVA with Tukey's post hoc test. No asterisks indicate the absence of statistical significance.

Analysis of the TRY values of AMD4,11 production rate, conversion yield and batch productivity (Figure 4) completed the media comparison. The use of BRMC‐MixAA led to approximately 45% higher AMD4,11 production rate than BRMC‐Control in the first cycle, whereas BRMC‐w/o AA caused performance loss (Figure 4a). During the second cycle, the production rates of the BRMC‐MixAA and BRMC‐Control groups were not significantly different (*p* = 0.97). The conversion yield of BRMC‐MixAA was the highest (Figure 4b), albeit at a statistically weak confidence level (*p* = 0.99). No statistical difference was observed between BRMC‐MixAA and BRMC‐Control for the batch productivities (Figure 4c) or in the distribution of product amounts (Figure 4d). Essentially, the findings underscore that replacing YE with the group of top‐ranked AAs ensures the same performance level for a minimum of two cycles.

### 3.3 Efficacy of Pyruvate Feeding in Enhancing Fed‐Batch Performance to Produce AMD4,11

The six successfully tested AAs in the BRMC, namely Glu, Ala, Leu, Val, Ile and Gly, either shared the precursor pyruvate and glyceraldehyde‐3‐phosphate (G3P) or, like Glu, function as metabolic hubs (Table S3). Notably, pyruvate and G3P are glycolytic precursors to produce key intermediates in the MEP pathway, namely, IPP and DMAPP, respectively. Hence, pyruvate supplementation was tested in the fed‐batch mode using slow‐growing cells and three mass flow rates: 0.09 (F1), 0.18 (F2) and 0.27 (F3) g_Pyr_ h^−1^ (See Section 2.5.2). The biphasic fed‐batch mode was selected to ensure transferability to an industrial scenario.

**FIGURE 5:**
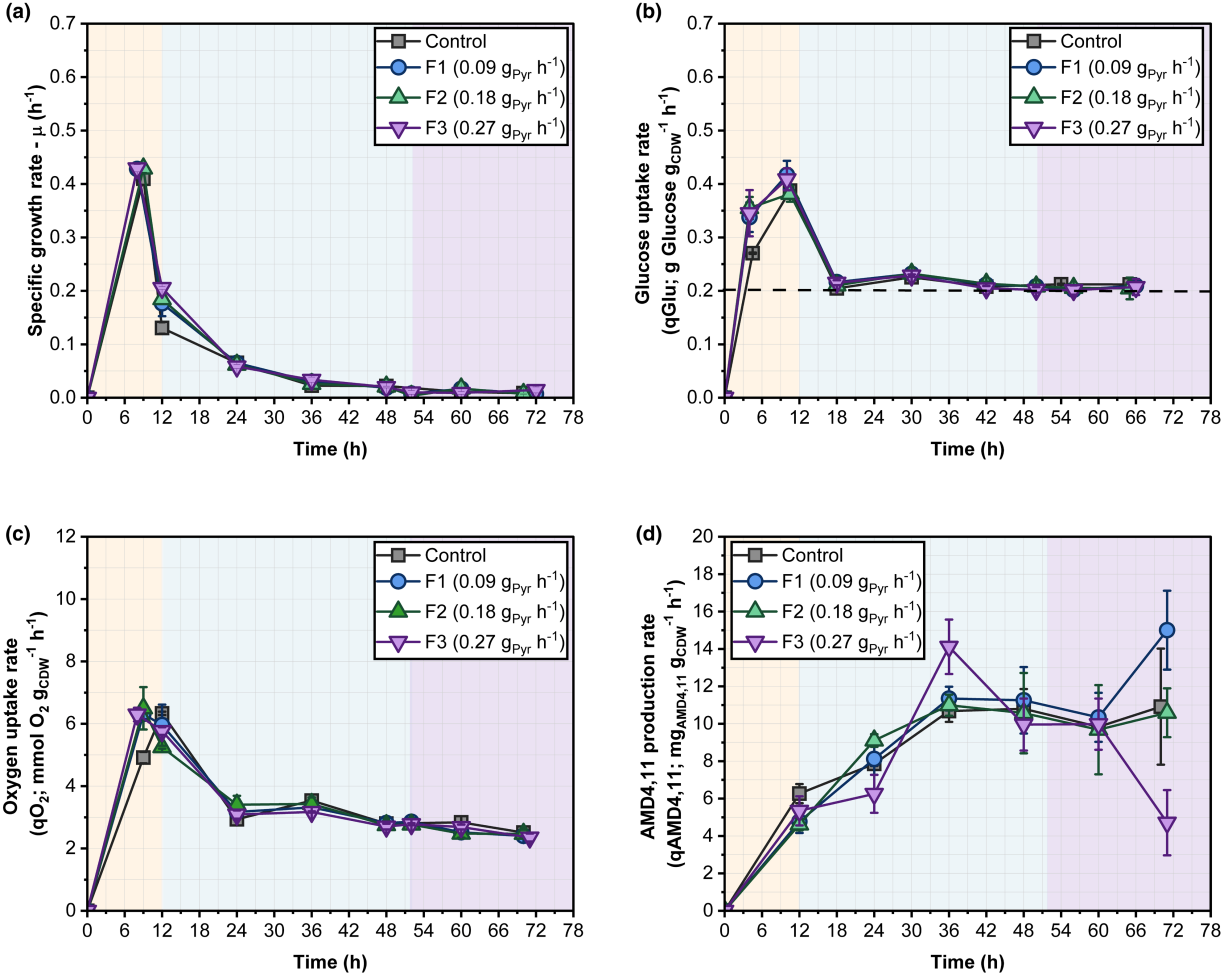
(a) Specific growth rate (μ; h^−1^), (b) glucose uptake rate (*q*_Glu_; g_Glucose_ g_CDW_^−1^ h^−1^), (c) specific oxygen uptake rate ($q_{O_{2}}$; mmol O_2_ g_CDW_^−1^ h^−1^), and (d) specific AMD4,11 production rate (*q*_AMD4,11_; mg_AMD4,11_ g_CDW_^−1^ h^−1^) with pyruvate supplementation during the stationary phase in a biphasic fed‐batch fermentation. The light yellow background indicates the batch phase, light blue indicates the fed‐batch phase, and light purple indicates the pyruvate supplementation phase. Data are presented as mean ± SD (*n* = 3).

As illustrated in Figure 5a–c, the rates of growth, glucose uptake and oxygen uptake showed similar trends regardless of pyruvate supplementation, which started after approximately 52 h. The mean values were 0.01 ± 0.00 h^−1^, 0.21 ± 0.00 g_Glucose_ g_CDW_^−1^ h^−1^ and 2.61 ± 0.18 mmol O_2_ g_CDW_^−1^ h^−1^, respectively. However, the onset of pyruvate feeding led to a maximum of 15.0 ± 2.1 mg_AMD4,11_ g_CDW_^−1^ with an optimum feed of 0.09 g_Pyr_ h^−1^ (F1). This was 37.5% higher than that of the control (Figure 5d).

**FIGURE 6:**
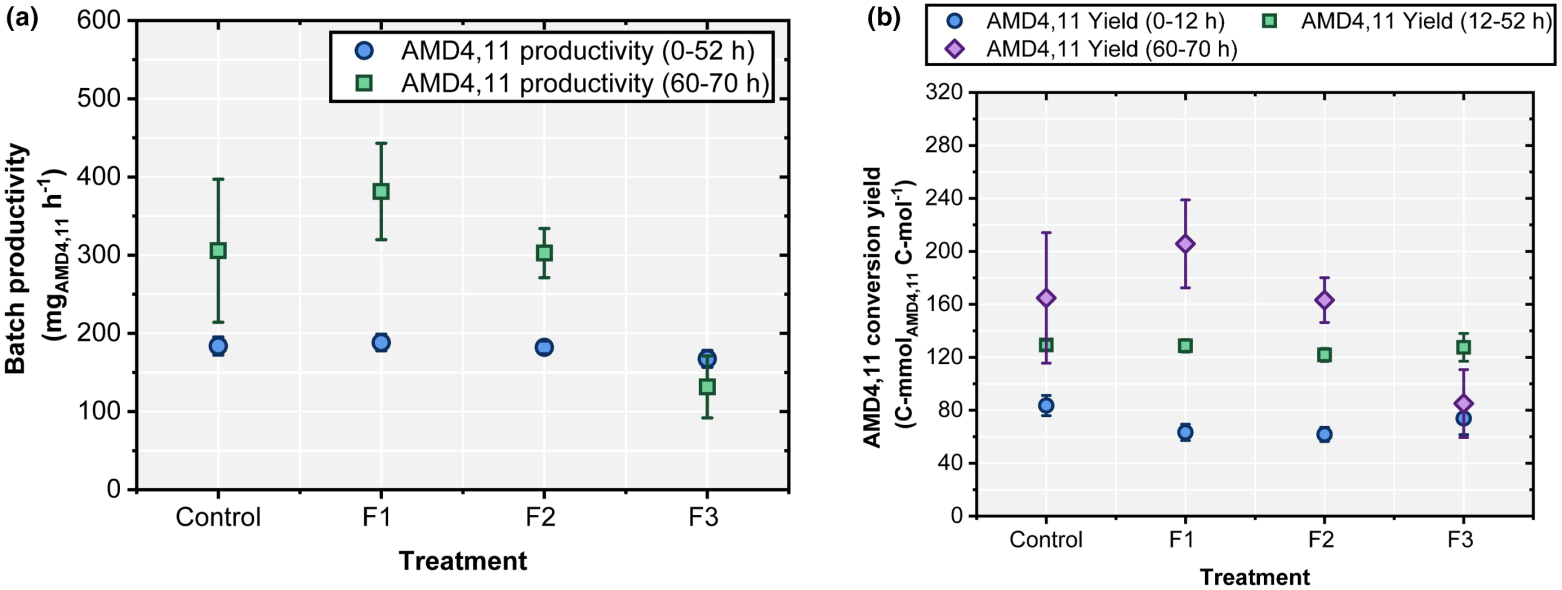
(a) Batch productivity (mg_AMD4,11_ h^−1^) and (b) amorpha‐4,11‐diene conversion yield (C‐mmol_AMD4,11_ C‐mol^−1^) with pyruvate supplementation during the stationary phase in a biphasic‐fed batch fermentation. Control (no pyruvate supplementation), F1 (0.09 g_Pyr_ h^−1^), F2 (0.18 g_Pyr_ h^−1^) and F3 (0.27 g_Pyr_ h^−1^). Data are presented as mean ± SD (*n* = 3).

The analysis of batch productivity and conversion yields revealed that all tests showed similar batch productivities of 32.03 ± 4.61 mg_AMD4,11_ h^−1^ before pyruvate feeding started (Figure 6a). Afterwards, however, proper pyruvate feeding with F1 and F2 significantly increased the performance (Figure 6b). Again, feeds F1 and F2 improved the substrate‐to‐product conversion yields, although their performances were similar. Notably, *Y*_PS_ was higher during the growth‐limited fed‐batch period than during the batch phase (see also Figure S4).

Pyruvate, glucose and oxygen uptake rates, along with the specific growth rate and AMD4,11 production rate (Table 1) from the pyruvate supplementation phase (60–70 h), were used as constraints for FBA analysis. This FBA enabled us to elucidate metabolic responses, identify tentative bottlenecks and examine resource allocation patterns specifically during pyruvate supplementation.

**TABLE 1:** Parameter definition for flux balance analysis constraints.

#	*q*_Pyr_ (mmol_Pyr_ g_CDW_^−1^ h^−1^)	*q*_Glu_ (mmol_Glucose_ g_CDW_^−1^ h^−1^)	$q_{O_{2}}$ (mmol O_2_ g_CDW_^−1^ h^−1^)	*μ* (h^−1^)	*q*_AMD4,11_ (mmol_AMD4,11_ g_CDW_^−1^ h^−1^)
Control	0.000	1.174	2.509	0.009	0.053
F1	0.038	1.160	2.403	0.007	0.073
F2	0.077	1.133	2.471	0.007	0.052
F3	0.115	1.147	2.351	0.015	0.023

**FIGURE 7:**
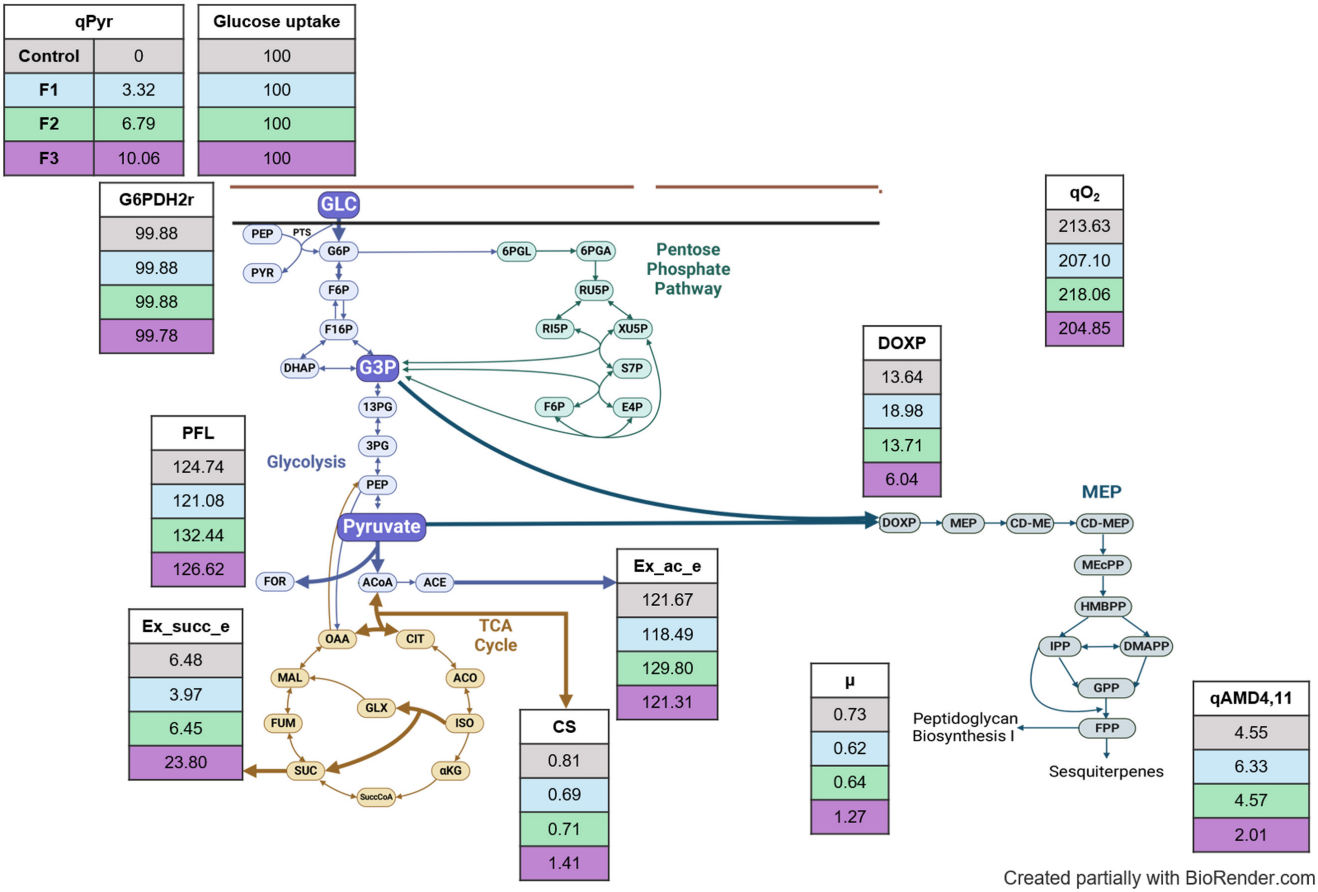
Flux balance analysis results of pyruvate supplementation experiments. All fluxes are in mmol g_CDW_^−1^ h^−1^ and were normalized by the specific glucose uptake rate. Glucose, oxygen ($q_{O_{2}}$) and pyruvate (*q*_Pyr_) uptake rates, specific growth rate (μ) and AMD4,11, production rate (*q*_AMD4,11_) were constraints of the FBA model. Nomenclature: Glucose‐6‐phosphate dehydrogenase (G6PDH2r), citrate dehydrogenase (CS), pyruvate formate‐lyase (PFL), succinate (Ex_succ_e) and acetate (Ex_ac_e) secretion, and 1‐deoxy‐D‐xylulose‐5‐phosphate synthase (DOXP). The highlighted lines represent the mentioned metabolic fluxes.

The set of selected fluxes involved in AMD4,11 production is shown in Figure 7. During the stationary phase, all carbon uptake by the cells in the control treatment originated from the glucose feed. In contrast, for F1, F2 and F3, 1.6%, 3.3% and 4.8% of the total carbon uptake derived from the pyruvate feed, respectively. Our FBA analysis revealed comparable fluxes via glucose‐6‐phosphate dehydrogenase (G6PDH2r) in all treatments, indicating consistent and inflexible carbon fluxes through glycolysis, even during pyruvate supplementation.

After the onset of pyruvate feeding F1 the following changes were observed: (1) the citrate dehydrogenase (CS) flux was 15% lower than the control, (2) the secretion of succinate (Ex_succ_e) and acetate (Ex_ac_e) were reduced by 38.7% and 2.6%, respectively, and (3) in contrast to F3, the rate of 1‐deoxy‐D‐xylulose‐5‐phosphate synthase (DOXP) was enhanced by 39.1%. No clear trends were observed in the pyruvate formate‐lyase flux. Production of organic acids and residual pyruvate in the fermentation broth was monitored through the fermentation (Figure S5).

**FIGURE 8:**
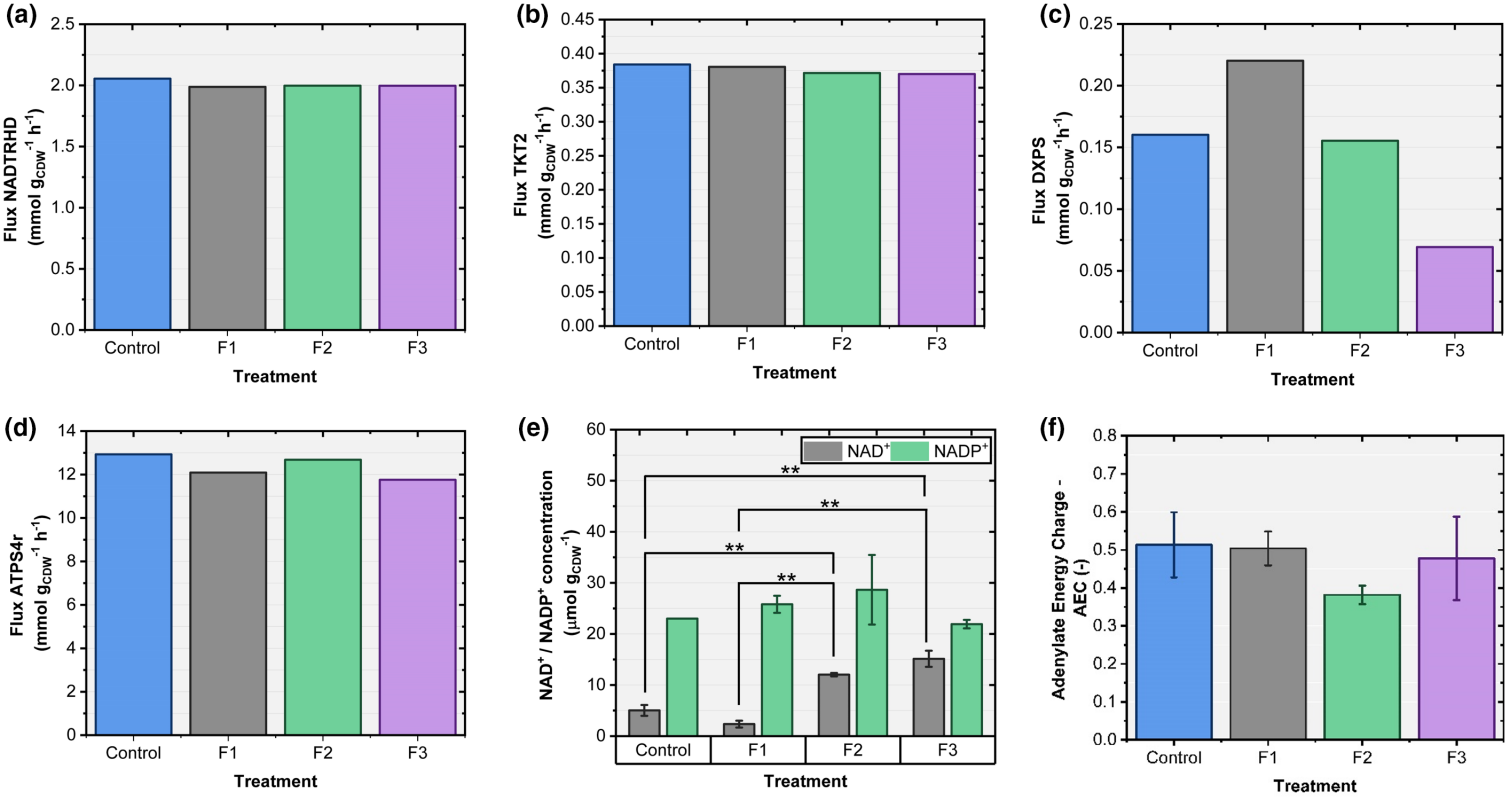
Flux balance analysis (FBA) results for flux through (a) NAD transhydrogenase (NADTRHD) converting NADPH to NADH, NADPH+NAD^+^ < − > NADP^+^ + NADH, (b) transketolase (TKT2) directing extra carbon back to glycolysis, (c) DOXP synthase (DXPS), (d) ATP synthase (ATPS4r) and (e) experimental values for NAD^+^ and NADP^+^, and (f) AEC values for sampling points used as constraints of the FBA. Data are presented as mean ± SE (*n* = 3). For (e, f), asterisks indicate statistically significant differences (*p ≤ 0.05; **p ≤ 0.01; ***p ≤ 0.001) within the groups as determined by ANOVA. No asterisks indicate the absence of statistical significance. Sample points for e and f corresponded to the time frame 60–70 h.

Further analysis regarding the supply of the reduction equivalents NADH and NADPH focused on transhydrogenase (NADTRHD) and transketolase (TKT; NADPH and NADH related). Additionally, intracellular concentrations of cofactors during pyruvate supplementation along with AEC were determined (Figure 8).

No changes were observed in the NADTRHD and TKT2 fluxes. Hence, there was no impact on transhydrogenase activity and no change in the carbon flux from the pentose phosphate pathway back to glycolysis (Figure 8a,b). As shown in Figure 8c, the DOXP synthase (DXPS) flux was improved for F1 compared with F2 and the control, whereas F3 exhibited lower fluxes. The mean flux via ATP synthesis remained in all treatments at 12.37 ± 0.54 mmol g_CDW_^−1^ h^−1^ (Figure 8d), which was also mimicked in the almost constant AEC values (See Figure S5 for detail information of AEC profiles). The NADP^+^ pool did not change during the tests compared with the control. However, the NAD^+^ pool showed remarkable differences compared with those of the control. Although they were approximately 3.7‐fold higher in F2 and F3, the pool was reduced after the onset of F1 (Figure 8e).

## 4 Discussion

The use of complex nutritional sources ensures high performance in terpene production, but introduces inherent variability, thereby impacting large‐scale reproducibility (Diederichs et al. 2014; Jacob, Hutzler, and Methner 2019; Jacob et al. 2019c; Tachibana, Watanabe, and Konishi 2019; Sparviero et al. 2023). Encouraging the adoption of synthetic media to reduce biomanufacturing costs further underscores the need to maintain key performance parameters including titer, specific production rate and conversion yield (TRY).

Biomass retention is the recommended standard for assessing strain performance at low specific growth rates, enabling the controlled transition of cells to slow growth without compromising volumetric productivity. Thus, the identification of AA needs was achieved by applying a sequential procedure comprising an exponential batch, slow‐growth biomass retention and a subsequent continuous mode. Evaluation of slow‐growing cells revealed that Glu, Ala, Leu, Val, Ile and Gly had the highest uptake rates. Their supply via YE feeding effectively reinstalled AMD4,11 production capacity. Furthermore, the application of the sequential procedure enabled the substitution of YE not only with the aforementioned AAs but also by their common metabolic precursor pyruvate. The latter was the result of a systematic evaluation that was applicable only to sequential procedures. The TRY values for AMD4,11 production improved in the BRMC and fed‐batch modes.

The substitution of YE by a selected group of AAs may initiate follow‐up research. Although the replacement of YE by AAs is already a process improvement, constitutive studies may aim to reduce AA to a smaller set to further reduce nutritional costs and determine strain‐ and product‐specific needs. For instance, in our study, replacing yeast extract with the top‐ranked AA in a BRMC process reduced the production cost of media components by 35%. A detailed approximation of medium expenses can be found in Table S4. Studies on recombinant protein production in *E. coli* showed that Glu substituted YE to alleviate metabolic burden (Chiang et al. 2022). Glu is involved in exchange systems (Gln/Glu) that increase acid tolerance and regulate electrical membrane potential at high osmolarity (McLaggan et al. 1994; Lu et al. 2013). Hence, Glu is a promising additive for increasing strain robustness.

Transformed into pyruvate, Ala is not only reintegrated into upper glycolysis but may also serve as a substrate for various production pathways (Katsube, Ando, and Yoneyama 2019; Maser et al. 2020). For instance, 3‐hydroxipropionic acid (3‐HP) production in *E. coli* is enhanced by high levels of pyruvate derived from D‐Ala degradation. Simultaneously, an increase in the intracellular Glu/Ala ratio redirects the carbon flux towards 3‐HP synthesis (Chaves et al. 2022). This observation, together with our results, opens the door for future studies. Investigating the substitution of branched chain AAs, Leu, Val and Ile deserve consideration, because they are relatively costly (Yokota and Ikeda 2017; Neinast, Murashige, and Arany 2019).

Besides substituting AA needs, pyruvate may well serve as ‘performance booster’. Notably, our study revealed that only 1% carbon of additional pyruvate feeding was sufficient to boost AMD4,11 formation by 37%. Although not identified, we hypothesise that pyruvate feeding may downregulate intracellular pyruvate‐related reactions or reallocate exogenous pyruvate directly into the MEP pathway. Our findings align with previous studies on limonene (Du et al. 2014), linalool (Wang et al. 2023) and AMD4,11 production (Zhou et al. 2013) in prokaryotes. Similar benefits have been observed across various microbial platforms (Zhou et al. 2013; Cao et al. 2016). However, a key distinction of our approach lies in its focus on decoupled‐growth production, when pyruvate is continuously fed to resting cells. In contrast, earlier studies centred on coupled‐growth production within batch processes, treating pyruvate as a static medium component rather than employing dynamic feeding strategies. Alternatively, intracellular pyruvate supply may be enhanced by well‐equilibrated metabolic engineering measures. Conversely, addressing pyruvate‐related side reactions to prevent reduction in glucose uptake remains challenging (Moxley and Eiteman 2021).

Alternatively, studies may investigate the supply of short‐chain keto acids, which may serve as AA precursors. Candidates such as α‐ketoglutarate (αKG) and oxaloacetate (OAA) may be problematic because of their roles in nitrogen metabolism (Senior 1975; Sauer and Eikmanns 2005). Increased αKG pools inhibit PTS, reducing glucose uptake (Doucette et al. 2011). Conversely, OAA transforms into pyruvate and phosphoenolpyruvate under gluconeogenic conditions, which potentially limits its efficacy as a glucose feed supplement. Moreover, decarboxylation of either αKG or OAA in the TCA cycle is restricted because microaerobic conditions are preferred for terpene production. Additionally, G3P may serve as an amino precursor in the feed. However, further metabolism may require elevated G3P dehydrogenase (GAPDH) activity, which likely conflicts with the regulation of other glycolytic enzymes (Cho et al. 2012).

Regarding the optimum pyruvate feeding, our FBA provided useful insights into maximizing the impact on product formation. For instance, higher pyruvate feed rates failed to enhance production capacity owing to an NADH/NAD^+^ imbalance. Thus, cells redirected carbon flux towards succinate production to increase NAD^+^ pools (See Figure S5). Multiple options may be considered to restore this balance, such as overexpression of nicotinic acid phosphoribosyltransferase (*pncB*) or reduction in the expression levels of NAD transhydrogenase (*udhA*) to avoid NADPH oxidation into NADH (Auriol et al. 2011; Liang et al. 2012). In addition, DOXP flux into the MEP pathway may be enhanced by pyruvate addition on slow‐growing cells, offering an alternative for process development to the traditional metabolic engineering strategy of DXPS overexpression (Banerjee and Sharkey 2014; Zhang and Hong 2020; Rinaldi, Ferraz, and Scrutton 2022). However, MEcPP flux, a common bottleneck in the production of isoprenoid precursors via the MEP pathway, needs to be estimated and adjusted to avoid the accumulation of toxic intermediates (Patil et al. 2021).

## 5 Conclusion

This study demonstrated that YE enhances AMD4,11 production in *E. coli* . The proposed sequential procedure revealed the need for AAs under slow‐growth conditions, highlighting pyruvate as a common precursor. Notably, this operation mode is most favoured for AMD4,11 production. Substituting YE with the top‐ranked AAs in a semi‐continuous process (BRMC) or implementing pyruvate feed during fed‐batch cultivation fostered microbial growth and maximum strain performance. Flux balance analysis elucidated the enhancement mechanism, emphasizing the increased carbon flux through DOXP, reduced oxidation byproducts and stable redox balance. Our results suggest the use of metabolic precursors such as pyruvate to boost cellular efficiency in the uncoupled growth production of secondary metabolites dependent on glycolytic intermediates. Furthermore, the lessons learned in this study can be applied to similar applications. In particular, optimizing slow‐growth product formation is a promising field of application that, despite its importance in real production scenarios, has not yet gained equal scientific interest.

## Author Contributions

**Carlos Castillo‐Saldarriaga:** conceptualization, investigation, methodology, writing – original draft, writing – review and editing. **Christine N. S. Santos:** writing – review and editing. **Stephen Sarria:** writing – review and editing. **Parayil K. Ajikumar:** writing – review and editing. **Ralf Takors:** funding acquisition, resources, supervision, conceptualization, writing – review and editing.

## Conflicts of Interest

The authors declare that the findings of this manuscript are part of a patent application submitted by the privately owned company, Manus Bio (Massachusetts, USA). All authors are listed as investors.

## Supporting information


Figures S1‐S5.

Tables S1‐S4.

## Data Availability

The data sets supporting the results of this study are available in our DaRUS repository (https://doi.org/10.18419/darus‐4552). Contact the corresponding author for further information.
