# Supplementary material for: Substitute Yeast Extract While Maintaining Performance: Showcase Amorpha‐4,11‐Diene Production
Source: Microb Biotechnol. 2024 Nov 21;17(11):e70056. doi: 10.1111/1751-7915.70056 (PMC11580704; doi:10.1111/1751-7915.70056)
Supplement: Supplementary file 1 — Figures S1‐S5. [file MBT2-17-e70056-s001.pdf]

Supplemental Information - Figures

Substitute yeast extract while maintaining performance: showcase amorpha-4,11-diene production

Carlos Castillo-Saldarriaga[1] Christine N.S. Santos[2] Stephen Sarria[2] Parayil K. Ajikumar[2] Ralf Takors[1+]

[1]Institute of Biochemical Engineering, University of Stuttgart, Allmandring 31, Stuttgart, Germany
[2]ManusBio, 43 Foundry Ave #230, Waltham, MA, USA
[+]Corresponding Author: Ralf Takors, ralf.takors@ibvt.uni-stuttgart.de, Tel: +49-711-685-64535

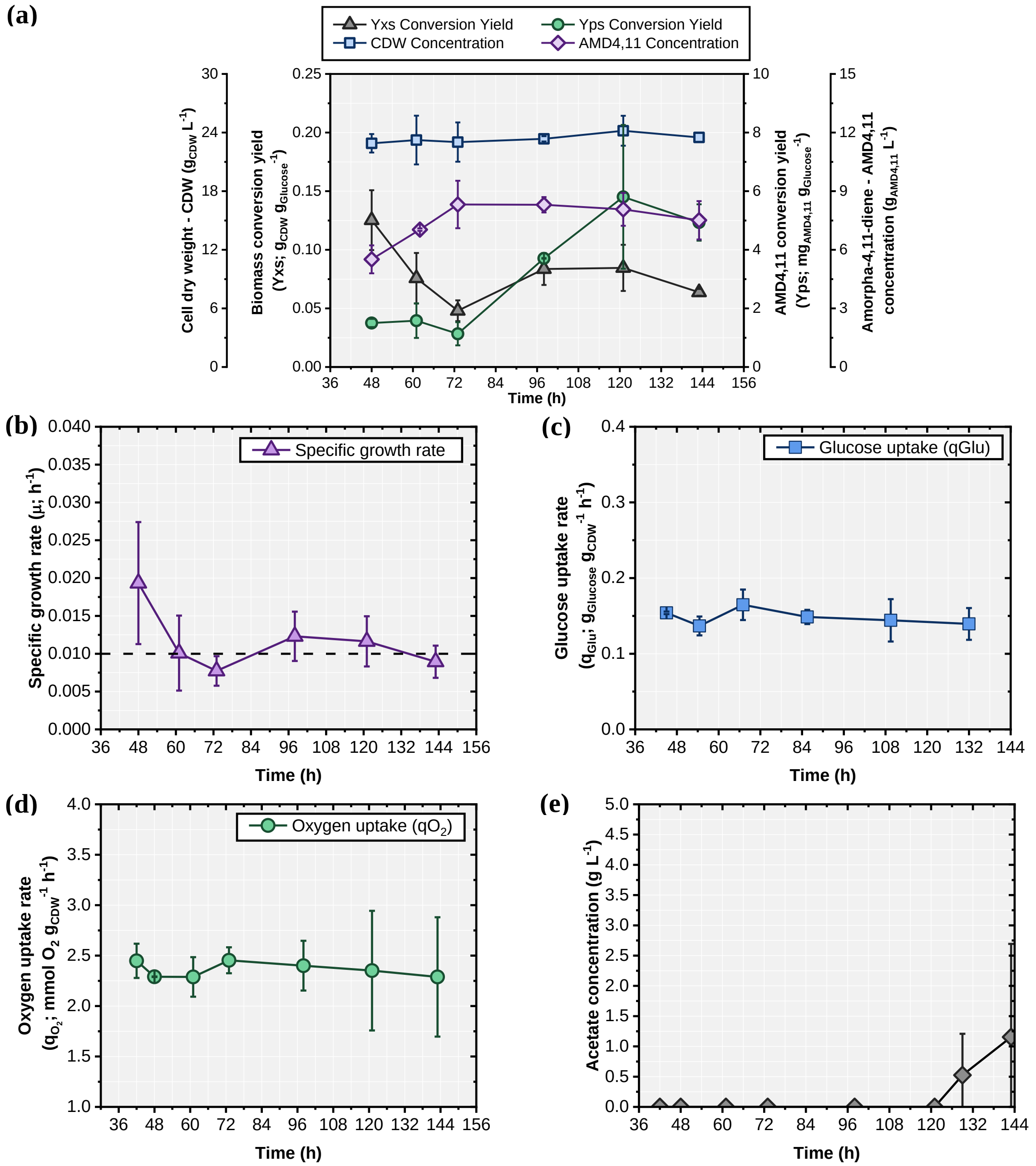

Figure S1. (a) Profiles of cell dry weight (CDW; g_{CDW} L^{-1}), conversion yield of glucose to biomass (Yxs; g_{CDW} $g_{Glucose}^{-1}$), conversion yield to amorpha-4,11-diene (Yps; $g_{AMD4,11}$ $g_{Glucose}^{-1}$), and amorpha-4,11-diene concentration (AMD4,11; $g_{AMD4,11}$ L^{-1}), **(b)** specific growth rate (μ; h^{-1}), **(c)** glucose uptake rate (q_{Glu}; $g_{Glucose}$ g_{CDW}^{-1} h^{-1}), **(d)** oxygen uptake rate (qO_2; mmol O_2 g_{CDW}^{-1} h^{-1}), and **(e)** acetate concentration (g L^{-1}) in a continuous stage of a sequential process with a dilution rate of 0.01 h^{-1}. Data are represented as mean ± SD (n=2).

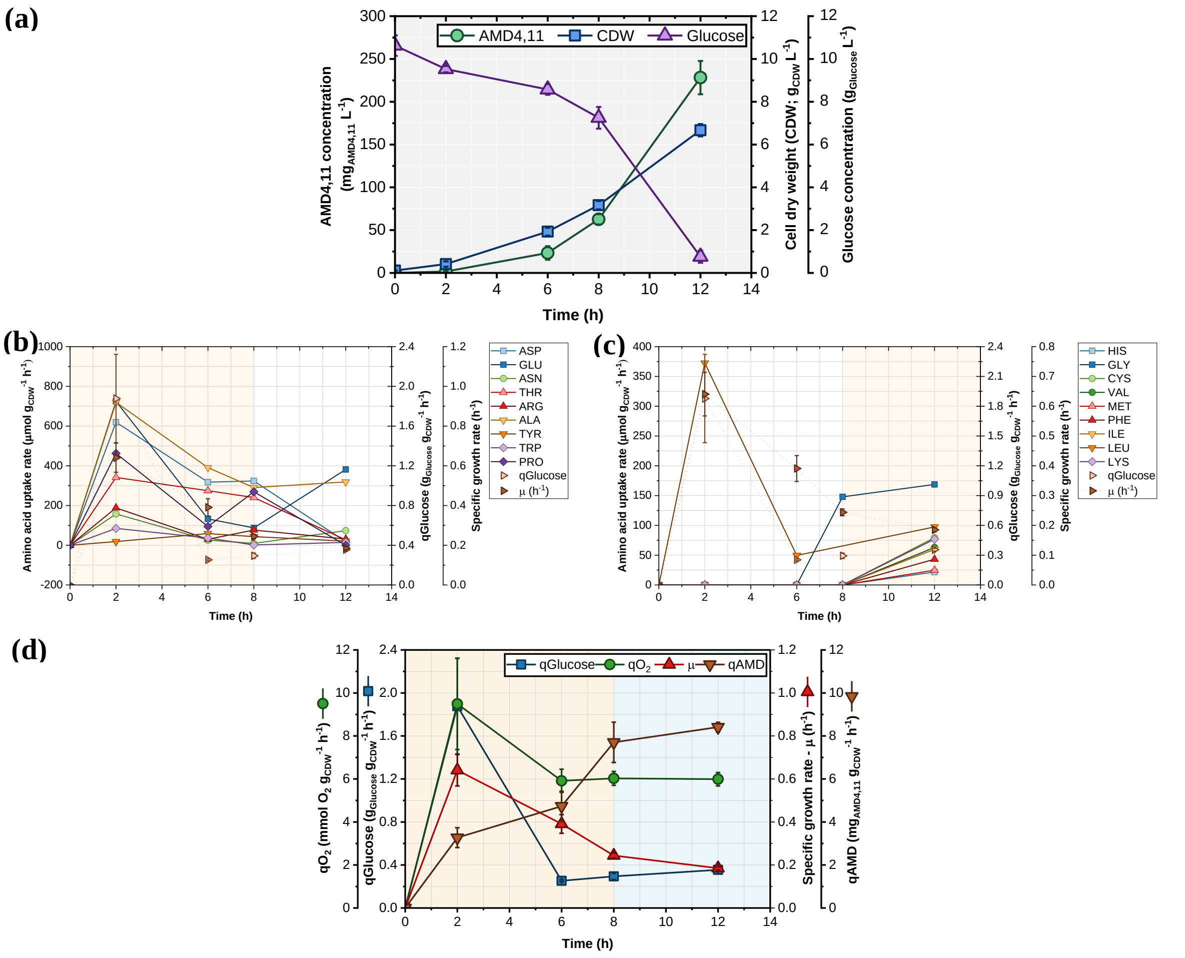

Figure S2. (a) Glucose, CDW and AMD4,11 concentrations, **(b)** first and **(c)** second group of AA concentrations, and **(d)** Glucose (q_{Glu}; $g_{Glucose}$ g_{CDW}^{-1} h^{-1}) and oxygen (q_{O2}; mmol O_2 g_{CDW}^{-1} h^{-1}) uptake rates, specific growth rate (μ; h^{-1}) and AMD4,11 production rate (q_{AMD}; $mg_{AMD4,11}$ g_{CDW}^{-1} h^{-1}) during the batch stage of a sequential process. Data are represented as mean ± SD (n=4).

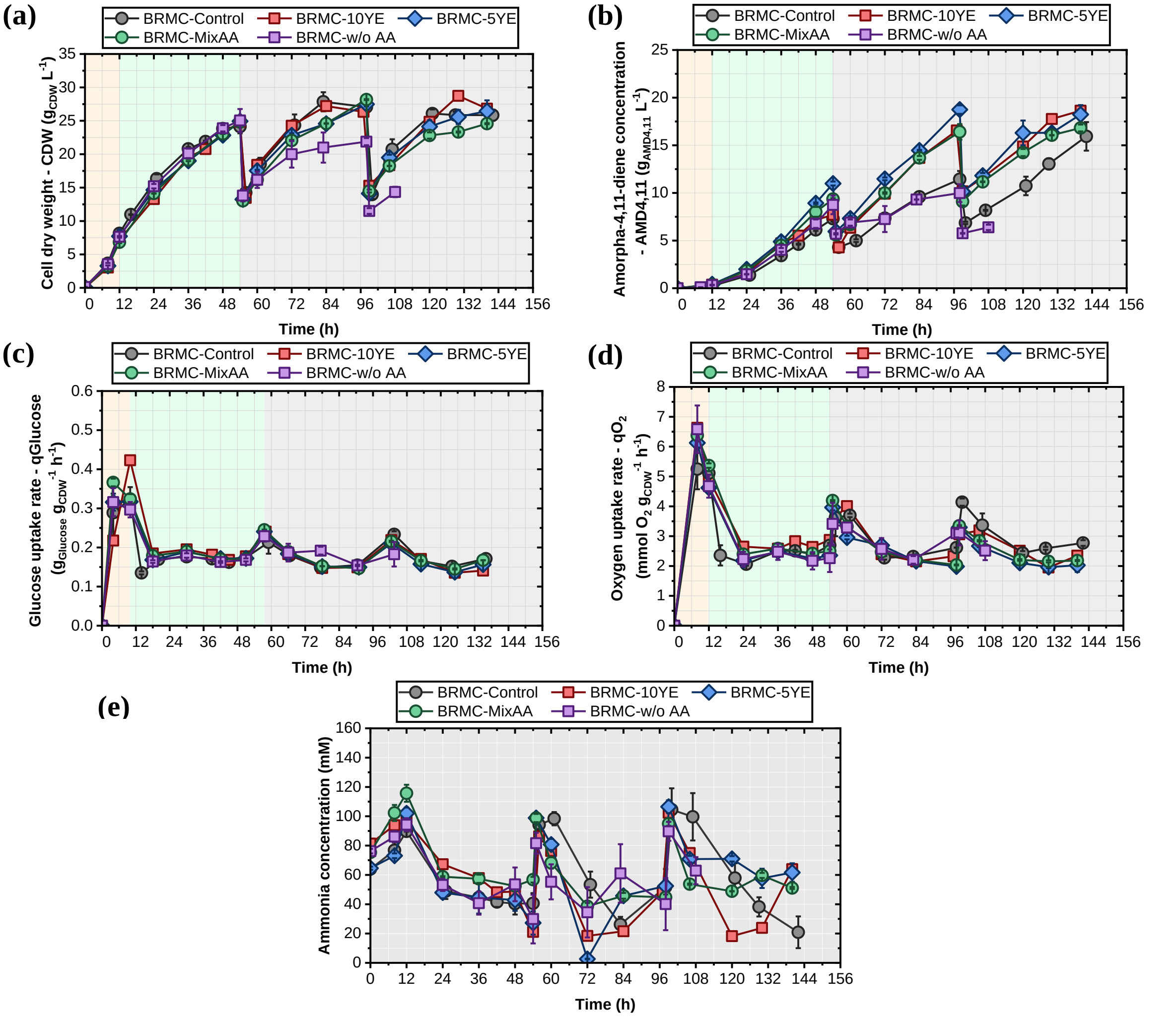

Figure S3. (a) Cell dry weight (CDW; g_{CDW} L^{-1}), **(b)** amorpha-4,11-diene concentration (AMD4,11; $g_{AMD4,11}$ L^{-1}), **(c)** glucose uptake rate (q_{Glu}; $g_{Glucose}$ g_{CDW}^{-1} h^{-1}) , **(d)** oxygen uptake rate (q_{O2}; mmol O_2 g_{CDW}^{-1} h^{-1}), **(d)** amorpha-4,11-diene production rate ($mg_{AMD4,11}$ g_{CDW}^{-1} h^{-1}), and **(e)** ammonia concentration (mM) during the fermentation course using a biomass retention by multiple cycles strategy (BRMC) using different replacement media. The light yellow background indicates the batch phase, light green indicates the biomass retention phase, and light grey multiple-cycle phase. Data are represented as mean ± SD (n=2).

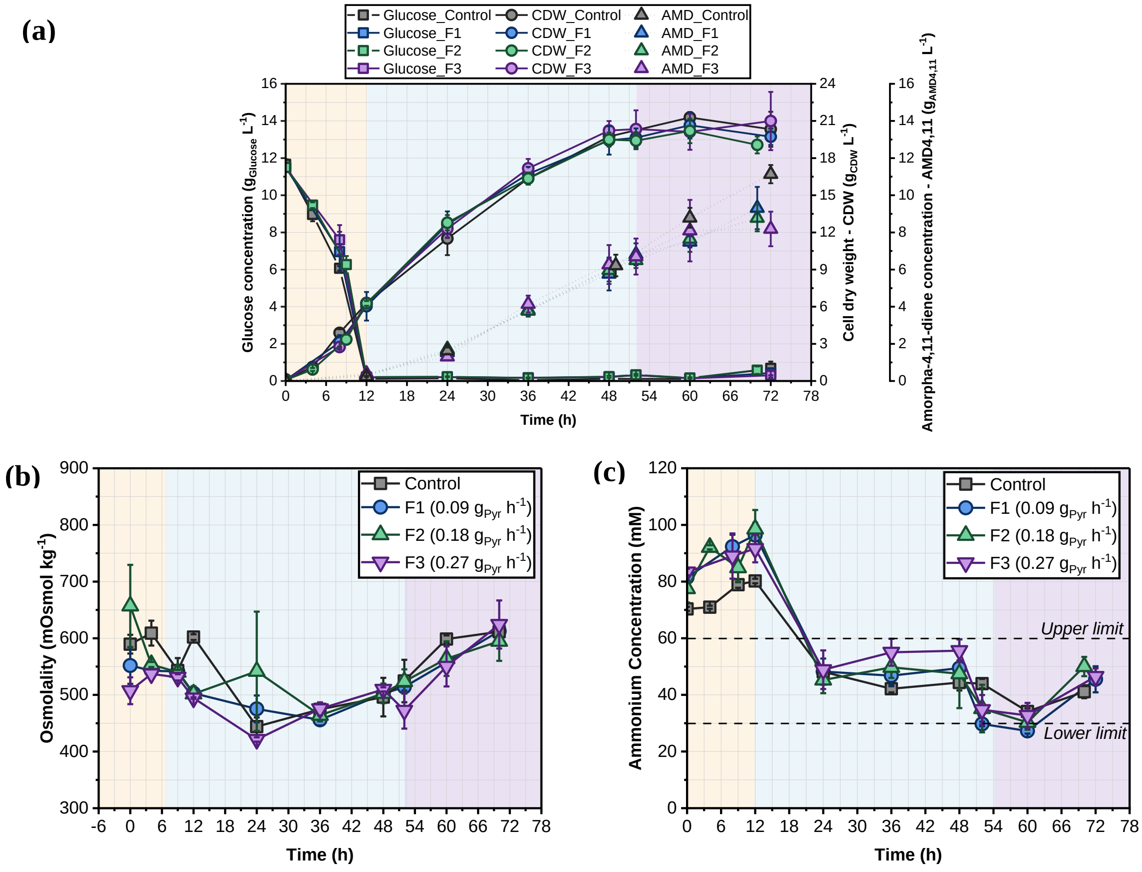

Figure S4. (**a**) Glucose, CDW and AMD4,11 concentrations, (**b**) osmolality and **(c)** ammonium concentration (mM), during a fed-batch process to produce AMD4,11 supplemented by different pyruvate mass flow rates during the stationary phase. Light yellow background indicates batch phase, and light blue indicates biomass retention phase. Data are represented as mean ± SD (n=3).

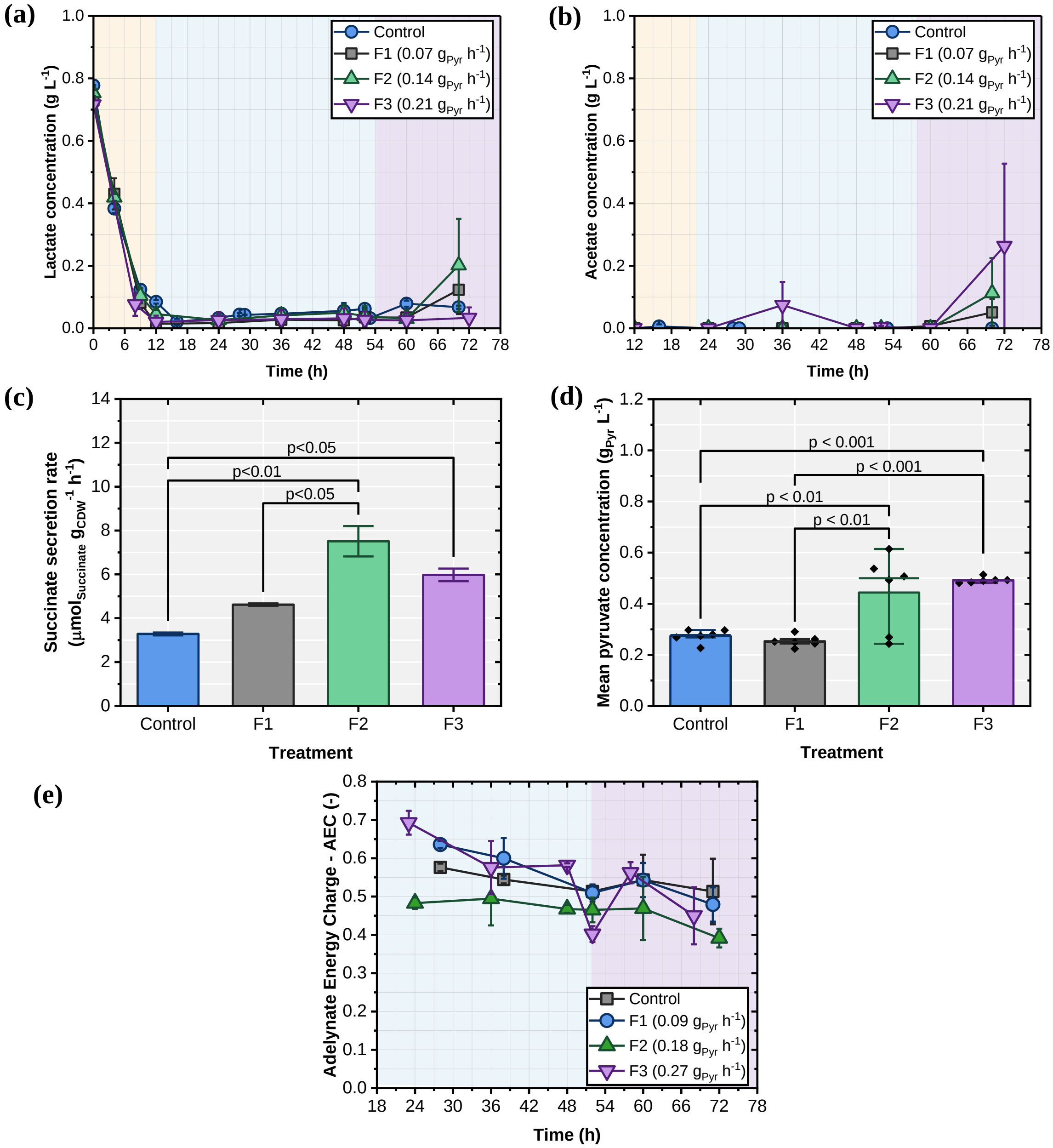

Figure S5. (a) Lactate concentration, **(b)** acetate concentration, **(c)** succinate secretion rate, **(d)** basal pyruvate concentration, and **(e)** Adelynate Energy Charge (AEC) with sodium pyruvate supplementation during the stationary phase in a biphasic fed-batch fermentation. For **(a)** and **(b)**, light yellow background indicates batch phase, light blue indicates fed-batch, and light purple indicates pyruvate supplementation phase. Data are represented as mean ± SD (n=3). For **(c)** and **(d)** one-way analysis of variance (ANOVA) with Tukey´s post hoc test at 70 h of fermentation. Data are represented as mean ± SD (n=2 and n=5). For **(e)** Data are represented as mean ± SD (n=3).
