# Supplementary material for: Substitute Yeast Extract While Maintaining Performance: Showcase Amorpha‐4,11‐Diene Production
Source: Microb Biotechnol. 2024 Nov 21;17(11):e70056. doi: 10.1111/1751-7915.70056 (PMC11580704; doi:10.1111/1751-7915.70056)
Supplement: Supplementary file 2 — Tables S1‐S4. [file MBT2-17-e70056-s002.pdf]

Supplemental Information -Tables

Substitute yeast extract while maintaining performance: showcase amorpha-4,11-diene production

Carlos Castillo-Saldarriaga[1] Christine N.S. Santos[2] Stephen Sarria[2] Parayil K. Ajikumar[2] Ralf Takors[1+]

[1]Institute of Biochemical Engineering, University of Stuttgart, Allmandring 31, Stuttgart, Germany
[2]ManusBio, 43 Foundry Ave #230, Waltham, MA, USA
[+]Corresponding Author: Ralf Takors, ralf.takors@ibvt.uni-stuttgart.de, Tel: +49-711-685-64535

Table S1. Yeast extract characterization.

Metabolite	Concentration (mg g^{-1} dry yeast extract)[a]
Aspartic acid (Asp)	21.83 ± 2.95 (13.53%)
Glutamic acid (Glu)	71.83 ± 4.77 (6.64%)
Asparagine (Asn)	15.33 ± 1.27 (8.28%)
Serine (Ser)	8.44 ± 0.95 (11.23%)
Glutamine (Gln)	3.60 ± 1.08 (29.86%)
Histidine (His)	7.82 ± 0.67 (8.54%)
Glycine (Gly)	13.10 ± 0.71 (5.41%)
Threonine (Thr)	18.38 ± 1.29 (7.04%)
Arginine (Arg)	10.41 ± 1.33 (12.79%)
Alanine (Ala)	45.03 ± 3.36 (7.46%)
Tyrosine (Tyr)	3.02 ± 0.00 (NA)
Cysteine (Cys)	39.68 ± 0.28 (0.71%)
Valine (Val)	25.75 ± 2.36 (9.15%)
Methionine (Met)	10.54 ± 0.85 (8.10%)
Tryptophane (Trp)	7.95 ± 1.26 (15.80%)
Phenylalanine (Phe)	21.81 ± 1.84 (8.44%)
Isoleucine (Ile)	27.33 ± 2.25 (8.24%)
Leucine (Leu)	45.51 ± 4.14 (9.11%)
Lysine (Lys)	28.58 ± 3.48 (12.17%)
Proline (Pro)	11.27 ± 1.08 (9.56%)

[a]As free amino acids in dried yeast extract. Coefficient of variation is reported in parenthesis. Data are represented as mean ± SD (n=6).

Metabolite	Concentration (mg g^{-1} dry yeast extract)[b]
Trehalose	42.28 ± 0.27 (0.64%)
Glucose	7.63 ± 0.04 (0.58%)
Succinate	5.48 ± 0.08 (1.51%)
Lactate	3.36 ± 0.10 (3.11%)
Formate	4.39 ± 1.20 (27.31%)
Acetate	4.63 ± 0.12 (2.69%)
Ethanol	3.19 ± 0.64 (19.92%)

[b]As free organic acids in dried yeast extract. Coefficient of variation is reported in parenthesis. Data are represented as mean ± SD (n=2).

Table S1. Yeast extract characterization (Continuation).

Proximate analysis	Concentration (% w w^{-1})
Water content	5.05 ± 0.05
Ash (575ºC)	11.48 ± 0.04

Macrominerals	Concentration (mg kg^{-1})
Sodium (Na)	1085 ± 7
Calcium (Ca)	484 ± 7
Magnesium (Mg)	742 ± 11
Potassium (K)	58800 ± 3111
Phosphorus (P)	12300 ± 141

Microminerals	Concentration (mg kg^{-1})
Aluminum (Al)	1.54 ± 0.60
Iron (Fe)	37.25 ± 1.34
Copper (Cu)	0.78 ± 0.13
Manganese (Mn)	1.26 ± 0.06
Zinc (Zn)	102 ± 1
Silicon (Si)	161 ± 10
Cobalt (Co)	2.67 ± 0.00
Molybdenum (Mo)	0.45 ± 0.04
Sulfur (S)	8590 ± 28
Selenium (Se)	2.10 ± 0.01
Lean (Pb)	0.21 ± 0.01
Nickel (Ni)	0.11 ± 0.13
Arsenic (As)	0.12 ± 0.06
Cadmium (Cd)	0.02 ± 0.00

Data are represented as mean ± SD (n=2).

Table S2. Replacement media composition for the evaluation of the BRMC

	BRMC-10 YE	BRMC-5YE	BRMC-MixAA	BRMC-w/o AA
**Component**	**Concentration (g L^{-1})**			
$(NH_4)_2SO_4$	4	4	4	4
KH_2PO_4	13.33	13.33	13.33	13.33
K_2HPO_4	5.09	5.09	5.09	5.09
Citric acid	1.11	1.11	1.11	1.11
Yeast extract	10	5	-	-
	**Concentration (mg L^{-1})**			
L-Alanine	1001	1001	1001	-
L-Glutamic acid	1596	1596	1596	-
L-Glycine	291	291	291	-
L-Leucine	1011	1011	1011	-
L-Valine	572	572	572	-
L-Isoleucine	607	607	607	-
$FeSO_47H_2O$	44.47	44.47	44.47	44.47
$MgSO_4$	334	334	334	334
Thiamine hydrochloride	5	5	5	5
	**Concentration (mL L^{-1})**			
Trace elements solution[a]	1.22	1.22	1.22	1.22
Struktol® J647 (Antifoam)	0.61	0.61	0.61	0.61

[a]Trace elements solution composition can be found in the material and methods section.

Table S3. Physicochemical characteristics of the amino acids detected in the yeast extract.

Amino acid	Metabolic precursors	Energetic cost (~P)[b]	NADH[c]	NADPH[c]	ATP consumption[c]	Hydrophobicity Index[d]	AAs in *E. coli* composition (%w w^{-1})[e]
Glu	α-ketoglutarate	15.3	0	1	2	Neutral	3.28
Ala	Pyruvate, AcetylCoA	11.7	0	1	2	Hydrophobic	3.55
Leu	Pyruvate	27.3	-1	2	2	Very hydrophobic	4.95
Val	Pyruvate, Threonine (Oxaloacetate [TCA cycle]), Glutamic acid (α-ketoglutarate)	23.3	0	2	4	Very hydrophobic	4.08
Ile	Serine (Glyceraldehyde-3-phosphate [G3P])	32.3	0	5	10	Very hydrophobic	3.19
Gly	Serine (Glyceraldehyde-3-phosphate [G3P])	11.7	-1	1	0	Neutral	3.40
Cys	Glyceraldehyde-3-phosphate (G3P)	24.7	-1	3	10	Hydrophobic	0.92
Asp	Oxaloacetate (TCA cycle)	24.7	0	1	2	Neutral	2.67
Lys	Aspartic acid (Oxaloacetate [TCA cycle]) Pyruvate	12.7	0	4	10	Hydrophilic	4.31
Thr	Aspartic acid (Oxaloacetate [TCA cycle])	30.3	0	3	8	Neutral	2.49
Phe	Pyruvate – Phosphoenolpyruvate (Shikimate pathway)/D-erythrose 4 phosphate (PPP)	18.7	0	2	5	Very hydrophobic	2.65
Pro	Glutamic acid (α-ketoglutarate)	52	0	3	7	Hydrophilic	2.09
Asn	Aspartic acid (Oxaloacetate [TCA cycle])	20.3	0	1	4	Hydrophilic	2.67
Ser	Glyceraldehyde-3-phosphate (G3P)/Glutamic acid	14.7	-1	1	0	Neutral	1.83
Arg	Glutamic acid (α-ketoglutarate)	11.7	-1	3	9	Hydrophilic	4.52
Trp	Pyruvate – Phosphoenolpyruvate (Shikimate pathway)/D-erythrose 4 phosphate (PPP)	27.3	-1	1	5	Very hydrophobic	1.03
Tyr	Pyruvate – Phosphoenolpyruvate (Shikimate pathway)/D-erythrose 4 phosphate (PPP)	74.3	-1	2	3	Hydrophobic	2.19
His	5-phospho-α-D-ribose-1-diphosphate (PPP)	50	-2	2	6	Neutral	1.26
Met	Oxaloacetate [TCA cycle]/Cysteine	34.3	-1	6	20	Very hydrophobic	1.96

[a]Abbreviations for amino acids (AAs): Glu, Glutamic acid (Glutamate); Ala, Alanine; Leu, Leucine; Val, Valine; Ile, Isoleucine; Gly, Glycine; Asp, Aspartic acid (Aspartate); Lys, Lysine; Thr, Threonine; Phe, Phenylalanine; Pro, Proline; Asn; Asparagine; Ser, Serine; Arg, Arginine; Trp, Tryptophan; Tyr, Tyrosine; His, Histidine; Cys, Cysteine; Met, Methionine.

[b] (Akashi and Gojobori, 2002).

[c] (Kaleta *et al.*, 2013).

[d] (Sigma-Aldrich, 2023)

[e] (Feist *et al.*, 2007)

Table S4. Approximation of the fermentation media cost.

A market search was carried out to determine the current prices of yeast extract from various suppliers.

Table S4a. Comparison of the yeast extract NuCel® 851 MG price respect to other yeast extracts available in the market.

Stock unit (kg)	Price (USD)	Price per mass unit (USD kg^{-1})	Reference	Supplier	Source	Date
20	110	5.50	FM903	Angel Yeast	https://www.tradeindia.com/products/angel-yeast-extract-powder-fm903-for-fermentation-industry-8020687.html	12/06/2024
25	689.08	27.56	15179323	ThermoScientific	https://www.fishersci.de/shop/products/yeast-extract-29/15179323#Yeast%20Extract	12/06/2024
20	585	29.25	NuCel ® 851 MG	Procelys by Lesaffre	Internal communication	11/06/2024
15	774.92	51.66	HY-YEST 412	Kerry	https://www.fishersci.com/us/en/catalog/search/products?keyword=KERRY+yeast+extract	12/06/2024
10	587.53	58.75	YEA10	Formedium®	https://formedium.com/product/yeast-extract-powder/	12/06/2024
10	829.46	82.95	2363.7	Carl Roth®	https://www.carlroth.com/de/de/extrakte/hefeextrakt/p/2363.7	12/06/2024
25	2625.5	105.02	Y20020-25000	RPI Research Products International	https://www.rpicorp.com/products/growth-media/powder/yeast-extract-powder-25-kg.html	12/06/2024
25	3454	138.16	RM027	Himedia	https://www.himediastore.com/yeast-extract-powder	12/06/2024
10	1594.3	159.43	J850-10KG	VWR® Life Science	https://de.vwr.com/store/product/en/8563485/vwr-yeast-extract	12/06/2024
11.3	3108.35	275.08	16239771	Gibco™	https://www.fishersci.de/shop/products/bd-bbl-dehydrated-culture-media-additive-yeast-extract-3/16239771#Yeast%20Extract	12/06/2024

The graph bellow represents the list of prices for different yeast extract suppliers. NuCel® 851 MG is represented in orange, while the rest of the yeast extracts are in blue. Upon comparing the prices, it was found that the YE used in our study has a competitive price compared to others although it is not the cheapest on the market.

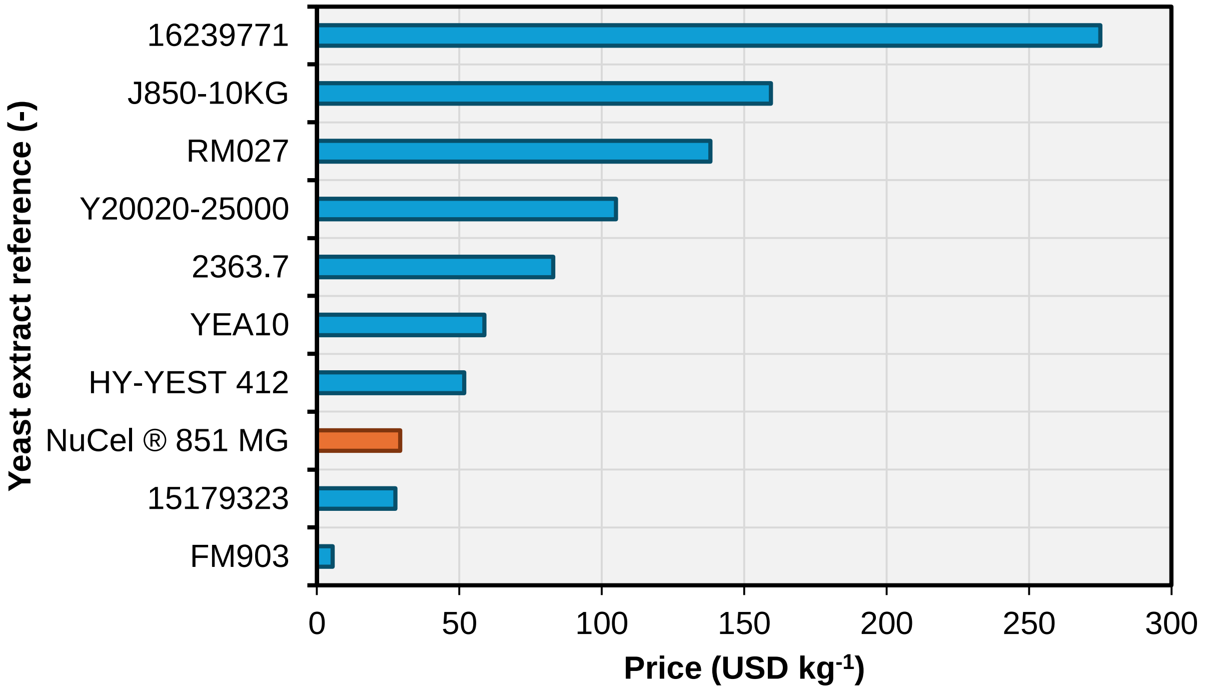

The market search also included prices for major media components, yeast extract, amino acids and minor media components.

Table S4b.Media component prices.

Major components of the fermentation media.

Component	Formula	Price (USD kg^{-1})	Reference	Supplier	Source	Date
Ammonium sulfate	$(NH4)_2SO_4$	13.63	000-52	Chem-Impex	https://www.chemicalbook.com/ProductList_En.aspx?cbn=CB9466357&c=100KG&left=True#J_Condition	12/06/2024
Potassium dihydrogen phosphate	KH_2PO_4	24.50	00-746	Chem-Impex	https://www.chemimpex.com/potassium-phosphate-monobasic-anhydrous	12/06/2024
di-Potassium hydrogen phosphate	K_2HPO_4	17.50	00-438	Chem-Impex	https://www.chemicalbook.com/ProductList_En.aspx?kwd=7758-11-4&c=100KG&left=True#J_Condition	12/06/2024
Citric acid	C6H8O7	10.50	02-107	Chem-Impex	https://www.chemicalbook.com/ProductList_En.aspx?kwd=citric%20acid&c=100KG&left=True#J_Condition	12/06/2024

Amino acids.

Aminoacid	Formula	Price (USD kg^{-1})	Reference	Supplier	Source	Date
Glutamic acid	C5H9NO4	2.63	N.A.	N.A.	https://catcost.chemcatbio.org/materials-library	11/06/2024
L-Alanine	C3H7NO2	50.86	000-17	Chem-Impex	https://www.chemimpex.com/dl-alanine	12/06/2024
L-Leucine	C6H13NO2	57.6	00-200	Chem-Impex	https://www.chemimpex.com/category/search/L-leucine/2?filter=&search=L-leucine&type=q&keywordoption=ANY&cid=2&fltrdesc=	12/06/2024
L-Valine	C5H11NO2	40.71	00-317	Chem-Impex	https://www.chemicalbook.com/ProductList_En.aspx?kwd=L-VALINE&c=100KG&left=True#J_Condition	12/06/2024
L-Isoleucine	C6H13NO2	102.2	00-263	Chem-Impex	https://www.chemimpex.com/l-isoleucine	12/06/2024
L-Glycine	C2H5NO2	18.05	00-163	Chem-Impex	https://www.chemicalbook.com/ProductList_En.aspx?cbn=CB5336487&c=100KG&left=True#J_Condition	12/06/2024

Yeast extract NuCel® 851 MG

Component	Formula	Price (USD kg^{-1})	Reference	Supplier	Source	Date
Yeast extract	-	29.25	NuCel ® 851 MG	Procelys by Lesaffre	Internal communication	11/06/2024

Minor components of the fermentation media.

Component	Formula	Price (USD kg^{-1})	Reference	Supplier	Source	Date
Iron (II) sulfate heptahydrate	$FeSO_4 \cdot 7H_2O$	13.23	3722.5	Carl Roth®	https://www.carlroth.com/de/de/von-a-bis-z/eisen%28ii%29-sulfat-heptahydrat/p/3722.5	11/06/2024
Magnesium sulfate	$MgSO_4$	15.52	1A99.4	Carl Roth®	https://www.carlroth.com/de/de/regenerierbare-trocknungsmittel/magnesiumsulfat/p/1a99.4	11/06/2024
Thiamine hydrochloride	$C_{12}H_{17}ClN_4OS \cdot HCl$	155.15	W332208	Sigma-aldrich	https://www.sigmaaldrich.com/DE/en/product/aldrich/w332208	12/06/2024

The cost of the fermentation media was estimated based on the market prices of the main components:

Table S4c.Cost of media fermentation based on market prices of main components.

	BRMC-Control		BRMC-MixAA		BRMC-w/o AA	
**Component**	**Concentration (g L^{-1})**	**Price (USD)**	**Concentration (g L^{-1})**	**Price (USD)**	**Concentration (g L^{-1})**	**Price (USD)**
Ammonium sulfate	4	0.054513	4	0.054513	4	0.054513
Potassium dihydrogen phosphate	13.33	0.326585	13.33	0.326585	13.33	0.326585
di-Potassium hydrogen phosphate	5.09	0.089075	5.09	0.089075	5.09	0.089075
Citric acid	1.11	0.011655	1.11	0.011655	1.11	0.011655
Yeast extract	20	0.585000	0	0.000000	0	0.000000
	**Subtotal**	1.066828	Subtotal	0.481828	Subtotal	0.481828
	**Concentration (mg L^{-1})**	**Price (USD)**	**Concentration (mg L^{-1})**	**Price (USD)**	**Concentration (mg L^{-1})**	**Price (USD)**
L-Glutamic acid	0	0.000000	1001	0.002633	0	0.000000
L-Alanine	0	0.000000	1596	0.081173	0	0.000000
L-Leucine	0	0.000000	291	0.016762	0	0.000000
L-Valine	0	0.000000	1011	0.041158	0	0.000000
L-Isoleucine	0	0.000000	572	0.058480	0	0.000000
L-Glycine	0	0.000000	607	0.010959	0	0.000000
	**Subtotal**	0.000000	Subtotal	0.211163	Subtotal	0.000000
	**Concentration (mg L^{-1})**	**Price (USD)**	**Concentration (mg L^{-1})**	**Price (USD)**	**Concentration (mg L^{-1})**	**Price (USD)**
Iron (II) sulfate heptahydrate	44.47	0.000588	44.47	0.000588	44.47	0.000588
Magnesium sulfate	334	0.005182	334	0.005182	334	0.005182
Thiamine hydrochloride	5	0.000776	5	0.000776	5	0.000776
	**Subtotal**	0.006546	Subtotal	0.006546	Subtotal	0.006546
	**Production cost**	**1.073374**	**Production cost**	**0.69954**	**Production cost**	0.48837

In the BRMC approach, different compositions of the exchange media were evaluated. The cost approximation for each media and the productivity are shown next.

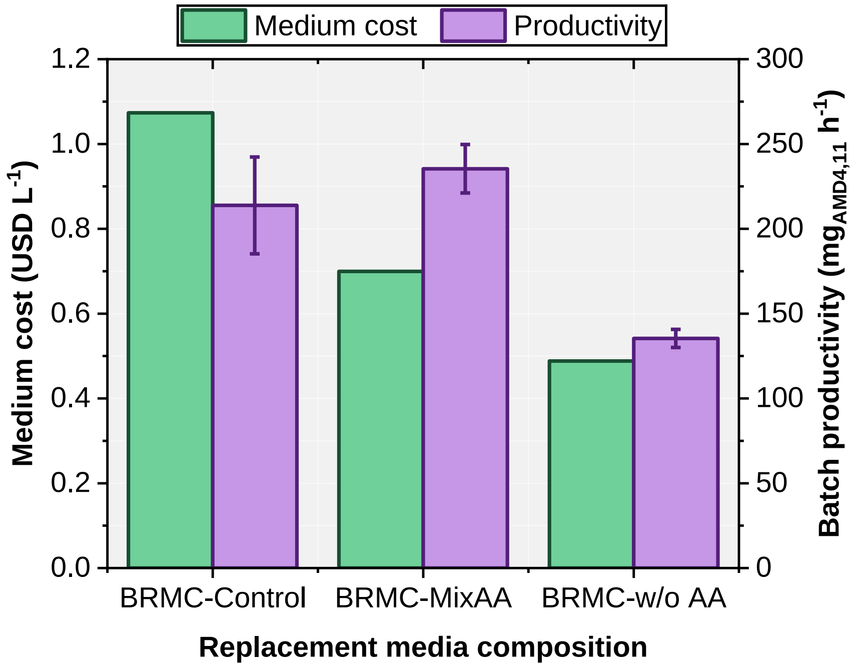

The productivity of AMD4,11 was successfully increased by replacing YE with the top-ranked AAs. Additionally, the cost of the fermentation media decreased from 1.07 to 0.7 USD L^{-1}, resulting in significant savings in operation expenses.

References

Akashi, H., and Gojobori, T. (2002) Metabolic efficiency and amino acid composition in the proteomes of Escherichia coli and Bacillus subtilis. *Proc Natl Acad Sci U S A* **99** (6): 3695–3700.

Feist, A.M., Henry, C.S., Reed, J.L., Krummenacker, M., Joyce, A.R., Karp, P.D., *et al.* (2007) A genome-scale metabolic reconstruction for Escherichia coli K-12 MG1655 that accounts for 1260 ORFs and thermodynamic information. *Mol Syst Biol* **3:** 121.

Kaleta, C., Schäuble, S., Rinas, U., and Schuster, S. (2013) Metabolic costs of amino acid and protein production in Escherichia coli. *Biotechnol J* **8** (9): 1105–1114.

Sigma-Aldrich (2023) Aminosäuren-Referenztabelle: Hydrophobizitatsindex fur Gewohnliche Aminosauren. [WWW document]. URL https://www.sigmaaldrich.com/DE/de/technical-documents/technical-article/protein-biology/protein-structural-analysis/amino-acid-reference-chart.
